# Comprehensive Analysis of the Tumor Microenvironment in Cutaneous Melanoma associated with Immune Infiltration

**DOI:** 10.7150/jca.44413

**Published:** 2020-04-06

**Authors:** Pan Wang, Xinyu Zhang, Nan Sun, Zhihong Zhao, Jie He

**Affiliations:** 1 Department of Thoracic Surgery, National Cancer Center/National Clinical Research Center for Cancer/Cancer Hospital, Chinese Academy of Medical Sciences and Peking Union Medical College, Beijing 100021, China; 2 Department of body contouring and liposuction center, Plastic Surgery Hospital, Chinese Academy of Medical Sciences and Peking Union Medical College, Beijing 100144, China

**Keywords:** cutaneous melanoma, tumor microenvironment, immune infiltration, competing endogenous RNA, prognosis

## Abstract

Accumulating evidence suggests that the malignant phenotypes of cancers are determined not only by the intrinsic properties of cancer cells but also by components in the tumor microenvironment (TME). In this study, we comprehensively characterized the TME of cutaneous melanoma (CM). As a result, tumor stage, tissue site, ulceration, thickness as well as patient age, sex were associated with immune infiltration. Patients of higher immune infiltration exhibited better survival outcomes, and antitumor effector cells, such as CD8 T cells and M1 macrophages, were found in significantly higher numbers in those tissues. Differential expression of mRNAs and long non-coding RNAs (lncRNAs) was analyzed and utilized to construct an immune-related competing endogenous RNA network, in which a lncRNA-associated subnetwork that could positively regulate the expression of IFN-γ was highlighted. Functional analysis confirmed that this network was remarkably enriched in functional terms related to both immune response and tumor-intrinsic pathways. Finally, a total of 109 high-confidence prognostic genes were identified, and a gene module that contained several key immune checkpoint molecules or modulators (PD-1, PD-L1, PD-L2, and LCK) was screened, which confers survival benefit for CM patients as supported by both overall and relapse-free survival rates from different datasets.

## Introduction

Cutaneous melanoma (CM) is the most common type of malignancy arising from melanocytes. It is also the most aggressive skin cancer that causes approximately 60,700 deaths worldwide annually, accounting for more than 80% of skin cancer-related deaths 1. For most limited-stage melanomas, surgical resection of the primary tumor is a standard and successful treatment, while for extensive-stage cases, treatment modalities are more complicated because most single or even combination therapies are effective in only a subset of patients 2. Despite the encouraging clinical results of novel therapies, the prognosis of advanced cases remains unsatisfactory with the 5-year survival rate slightly above 20% 3.

Over the past decade, there has been increasing interest in understanding the roles of the immune system in the initiation and progression of cancer. Tumor microenvironment (TME) has become the focus of attention because it consists of both cancer cells and nonmalignant stromal cells, including various types of immune cells. The activation state and components of tumor-infiltrating immune cells are important parameters that influence tumor biology and predict tumor prognosis. For example, cytotoxic CD8 T cells and CD4 helper T cells target antigenic tumor cells to prevent tumor growth 4. A high level of activated CD8 T cells is associated with prolonged patient survival time in many cancers, including CM 5. Conversely, tumor-associated macrophages, mast cells and neutrophil granulocytes exert effects on promoting tumor progression, and the intense infiltration of these cells generally indicates poor prognosis 5.

In addition, tumor-infiltrating immune cells serve as potential drug targets. Recently, a new therapeutic strategy, known as immune checkpoint blockade, has been developed, aiming at restraining the molecular interplay between tumor cells and immune cells. Immunotherapy with several checkpoint blockers targeting programmed cell death protein 1 (PD-1), PD-1 ligand (PD-L1), or cytotoxic T lymphocyte antigen-4 (CTLA-4) has resulted in significant improvements in the clinical outcomes of patients with various types of cancers including melanoma 6. However, not all CM patients benefit from these drugs. A subset of patients who initially respond to immunotherapy later develop disease progression, suggesting the existence of intrinsic immune resistance 2. In previous studies, PD-L1 expression, tumor mutation burden, neoantigen load and deficient DNA mismatch repair have been associated with the immunotherapeutic responsiveness of cancer 7-10. However, none of these features alone have been validated as sufficient predictors 11. More importantly, TME and infiltrating immune cells are unique to each cancer type; thus, studying changes of immune infiltration with the associated molecular expression and interactive features based on an individual basis is crucial in elucidating the mechanisms underlying immune evasion by melanoma and immune checkpoint blockades.

In this study, based on a large-scale computational analysis of expression profiles of 879 CM tissues from The Cancer Genome Atlas (TCGA), Gene Expression Omnibus (GEO) and ArrayExpress, we comprehensively characterized the tumor immune microenvironment of CM from three different levels: the tissue level, cellular level and molecular level. The relationship between immune infiltration and clinicopathological characteristics as well as immune cell composition was explored. Both protein-coding genes and long noncoding RNAs (lncRNAs) that were associated with immune functions were identified and incorporated for network analysis. By performing Kaplan-Meier survival analysis with the TCGA cohort and in an independent dataset, we eventually discovered an immune-related gene module that conferred prognostic benefit for CM patients. The results are expected to generate novel insights into the tumor immune microenvironment of CM and provide potential biomarkers for clinical use.

## Materials and Methods

### Data collection

The level 3 sequencing data of mRNA, lncRNA and miRNA of the CM tissues were obtained from the TCGA database via the TCGAbiolinks package 12. For long RNAs, both raw counts and fragments per kilobase million (FPKM) data were collected. The datasets contained 468 samples, one for each patient. For miRNAs, only raw count data were obtained, and the dataset contained 448 samples. The associated clinical information of the patients and genomic subtypes was retrieved from the UCSC Xena database 13. For validation, the processed expression meta-dataset of 194 CM samples was downloaded from ArrayExpress with E-MTAB-6697. Another dataset that contained 214 CM samples was downloaded from GEO with GSE65904 14, in which the outcome and relapse-free survival (RFS) time of the patients were incorporated. A log2 transformation was applied to the processed expression data before further analysis.

### ESTIMATE

The infiltration of noncancerous cells in tumor tissues was assessed by ESTIMATE based on expression profile of 141 immune-related genes 15. The stromal, immune and ESTIMATE scores of the tumor tissues from 25 cancer types used in this study were computed using the RNA-seq V2 data from TCGA 15. The immune scores of the CM samples from the E-MTAB-6697 and GSE65904 datasets were calculated from the normalized expression data using the R package ESTIMATE 15.

### CIBERSORT estimation

Normalized gene expression data were used to infer the relative proportions of 22 immune cell types using CIBERSORT 16. The FPKM data of 466 tumor samples (2 samples with no available immune scores were excluded from this analysis) from the TCGA, or the processed expression data from E-MTAB-6697 and GSE65904 were used as input and the LM22, which contained 547 genes that accurately differentiate 22 individual human hematopoietic cell types, was used as a reference gene signature. Permutations were set to 1,000. Only samples with a CIBERSORT P<0.05 were considered eligible for further analysis.

### Differential gene expression analysis

The differentially expressed mRNAs (DEmRNAs) and lncRNAs (DElncRNAs) were identified using edgeR 17, with |log2 fold changes| > 1 and FDR < 0.05 considered significant. Molecules with average read counts greater than 1 were included for this analysis.

### Construction of the competing endogenous RNA (ceRNA) network

The ceRNA network was constructed using the “gdcCEAnalysis” function of the GDCRNATools package 18: (1) the lncRNA and mRNA must share a significant number of miRNAs (hypergeometric test, P<0.05); (2) the expression of the lncRNA and mRNA must be positively correlated (Pearson correlation, P<0.05); and (3) those common miRNAs should play similar roles in regulating the expression of the lncRNA and mRNA (regulation similarity>0). The raw count data of both miRNA and long RNA molecules were normalized by the “gdcVoomNormalization” function. All DEmRNAs and DElncRNAs were kept for this analysis, together with 523 miRNAs whose average read counts were greater than 10. The interactions between miRNAs and mRNAs that are supported by strong experimental evidence were obtained from miRTarBase 19, we obtained 8,377 unique miRNA-mRNA interactions. For miRNA-lncRNA regulations, a total of 73,087 nonredundant miRNA-lncRNA interactions supported by experimental evidence were downloaded from LncBase 20.

### Enrichment analysis

The first 300 upregulated or downregulated genes identified from the differential expression analysis were used in the functional enrichment analysis with the plug-in software for Cytoscape 21, ClueGO v_2.5.4 22. The terms from Gene Ontology (GO), Kyoto Encyclopedia of Genes and Genomes (KEGG), WikiPathways and Reactome were analyzed. The GO term fusion option was selected. The significance value was set as P-value < 0.05, and the Bonferroni (two-sided hypergeometric test) was used as the multiple test correction. The threshold of the κ score that reflects the association between two terms was set at 0.4, and similar terms were given the same color. GO analysis of the ceRNA network and protein-protein interaction (PPI) module was performed using the web-based Enrichr tool 23, with the criterion of adjusted P-value<0.05.

### Statistical analysis

In this study, unpaired two-sided t-test or the Wilcoxon signed rank test was used to make statistical comparisons between two patient groups. For a comparison of more than two groups, one-way ANOVA was utilized. All tests were carried out using Prism 8.0 (GraphPad, San Diego, USA) or R (version 3.6.0, Auckland, NZ). A P-value less than 0.05 was considered statistically significant for all statistical analyses if not otherwise specified.

### Survival analysis

Kaplan-Meier survival analysis was performed using the R package “survival” 24. Patients with survival times less than 30 days were filtered out beforehand, and the remaining patients were classified into high and low groups based on the median statistics. Survival curves were plotted using the “survfit” function in the survival package, and the differences in survival curves for the two groups were analyzed with the log-rank method using the function “survdiff”.

### PPI network analysis

The PPI network was constructed using the web-based Search Tool for the Retrieval of Interacting Genes/Proteins (STRING) database 25. The PPI pairs with an interaction score>0.4 were considered as significant and used for construction of the network. The MCODE plug-in from Cytoscape was then used to extract modules from the PPI network with default parameters 26.

## Results

### Tissue-level immune infiltration in CM and its clinical relevance

**Figure 1:**
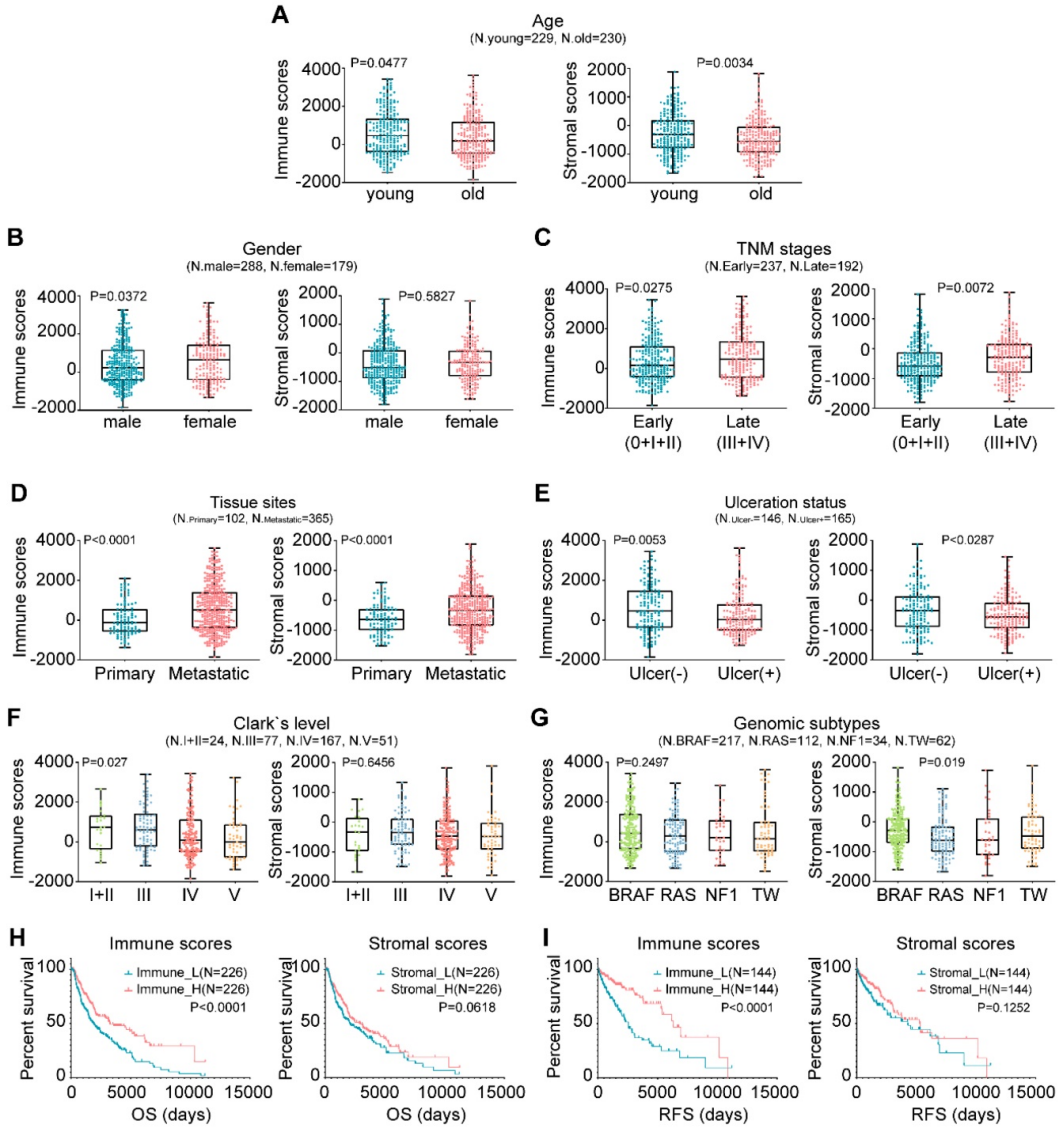
Associations between TME and clinicopathological features in CM. (A-G) Correlation of the immune and stromal scores with age, sex, TNM stage, tissue sites, ulceration status, Clark's level stage and genomic subtypes. (H-I) Correlation of the immune and stromal scores of CM tissues with the OS or RFS of the patients as illustrated by Kaplan-Meier survival curves. Patients were divided into two groups based on their median scores. P-values and the number of cases in each subgroup of the above analyses (A-I) are displayed. OS, overall survival; RFS, relapse-free survival; TW, triple-wild type.

In this study, the microenvironment of tumor tissues was assessed by the ESTIMATE algorithm based on two major types of the nontumor components, immune cells and stromal cells 15. Compared with other tumor types, the infiltration of noncancerous cells in CM was relatively prominent, particularly the immune cells, with average enrichment scores that ranked the 6th of all 25 evaluated cancers (Fig. S1). We next explored the correlation between the scores and clinicopathological characteristics. Briefly, younger patients generally had higher immune and stromal scores than older patients (Fig. 1A; P=0.0477 vs. P=0.0034). Female patients had higher immune and stromal scores than male patients, although the latter case was not statistically significant (Fig. 1B; P=0.0372 vs. P=0.5827). Extensive-stage cases exhibited significantly higher immune and stromal scores than early stage cases (Fig. 1C; P=0.0275 vs. P=0.0072), as did the tumors of metastatic sites compared with those of primary sites (Fig. 1D; P<0.0001). Tissues with ulceration or higher Clark levels had both lower immune and stromal scores (Figs. 1E-1F). As for genomic subtypes 27, the BRAF mutant cases had the highest stromal scores, followed by triple-wild type, NF1 mutant and RAS mutant cases (Fig. 1G; P=0.019). The immune scores of the BRAF subtype also ranked the highest of all four subtypes, although not statistically significant (P=0.2497). The association of immune infiltration with patient survival was analyzed. Kaplan-Meier survival curves showed that the cases with higher immune infiltration (immune_H) conferred prognostic benefit in both overall survival (OS) and RFS compared to those with lower immune infiltration (immune_L) (Figs. 1H-1I; P<0.0001), while no survival difference was observed between the samples with variable stromal infiltration (stromal_H and stromal_L). Collectively, these findings suggested that the TME, particularly immune cell infiltration, could have important effects on the pathogenesis of CM and clinical outcomes of patients.

### Characterization of the immune cell composition in CM

**Figure 2:**
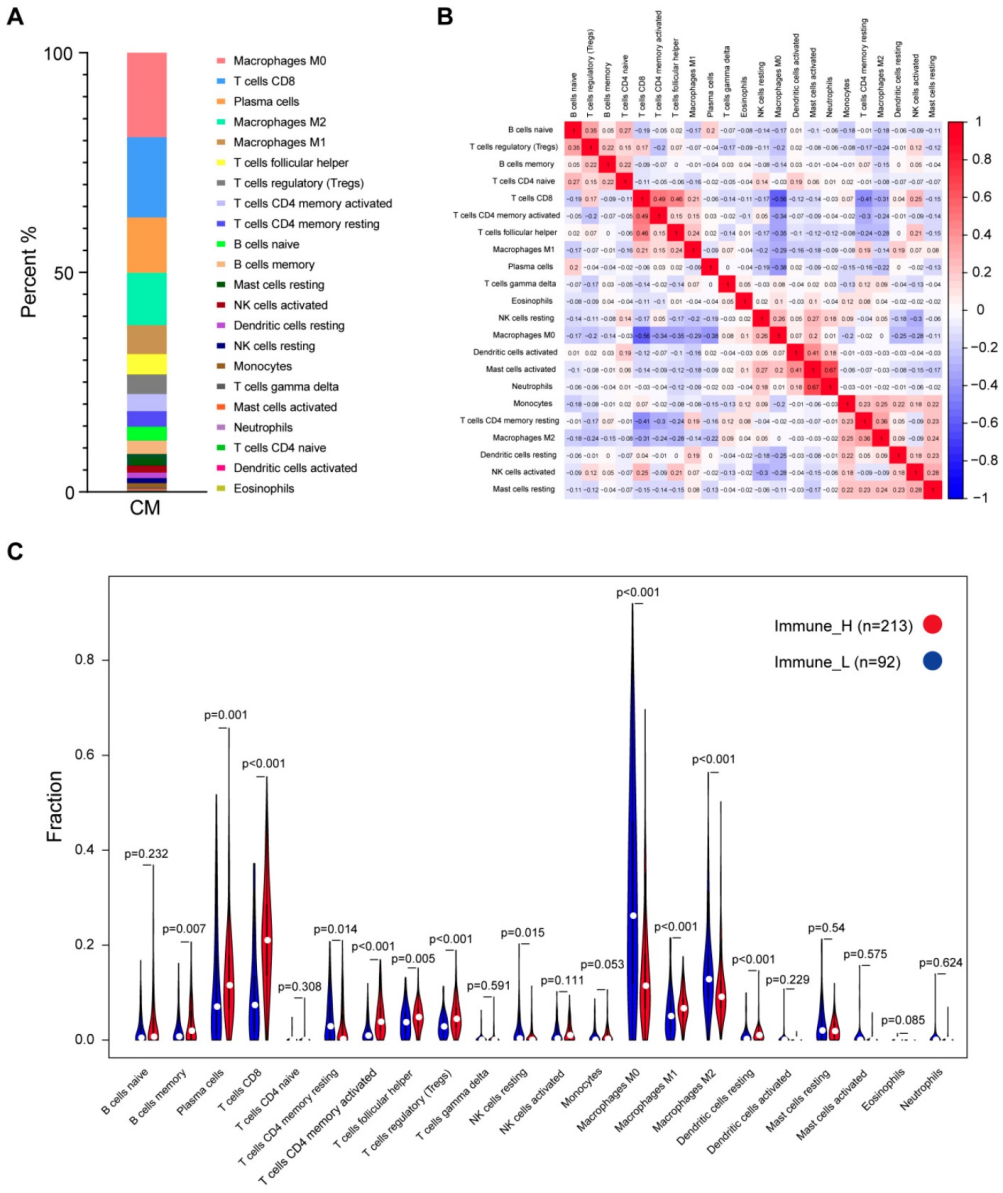
Distribution of immune cell fractions in CM tissues based on TCGA data. (A) The percentage of 22 types of immune cell subsets. (B) Correlation of 22 types of immune cell subsets. (C) Comparison of the immune cell fractions between tumor tissues of the immune_H and immune_L groups.

The CIBERSORT method was applied to characterize the cellular composition of the tumor-infiltrating immune cells in CM tissues 16. A total of 305 samples from the TCGA dataset were successfully deconvolved. Figure 2A shows that, on average, the M0 macrophages (19.22%) were the most abundant immune infiltrates, followed by CD8 T cells (18.29%), plasma cells (12.58%), M2 macrophages (11.95%) and M1 macrophages (6.58%). The same analysis was performed on two additional datasets, namely, GSE65904 and E-MTAB-6697, we found that the fractions of M0/M1/M2 macrophages and CD8 T cells were relatively stable across those datasets while some other cell fractions exhibited more variable, such as plasma cells, activated mast cells and neutrophils (Fig. S2), suggesting the heterogeneity of the immune infiltration of the TME. Correlation analysis based on the TCGA data suggested that the number of M0 macrophage cells was inversely related to that of CD8 T cells (r^2^=-0.56), plasma cells (r^2^=-0.38), follicular helper T cells (r^2^=-0.35), CD4 memory activated T cells (r^2^=-0.34) and M1 macrophage cells (r^2^=-0.29), indicating that functional antagonism might exist between M0 macrophages and those cells.

In contrast, a highly positive correlation between activated mast cells and neutrophils or activated dendritic cells (r^2^=0.67 or 0.41) suggested that these cells might function synergistically in tumor stroma (Fig. 2B). Comparison of the immune cell subsets between different immune infiltration levels revealed that CD8 T cells, activated memory CD4 T cells, plasma cells and M1 macrophages were present in significantly higher numbers in immune_H than in immune_L (P≤0.001, Wilcoxon signed rank test), while M0 and M2 macrophages were more abundant in the immune_L group (P<0.001; Fig. 2C). These results were largely confirmed in the ArrayExpress and GEO datasets (Figs. S3-S4). Kaplan-Meier analysis showed that naïve B cells were associated with shorter OS time, while M1 macrophages were associated with an improved outcome; Based on RFS, CD8 T cells, activated memory CD4 T cells and follicular helper T cells were correlated with longer survival time, whereas resting memory CD4 T cells indicated the opposite (P<0.05; Fig. S5). In summary, despite the variance of the immune cell composition from different studies, the antitumor immune activity of the immune_H group was enhanced.

### Association between molecular expression and immune infiltration

**Figure 3:**
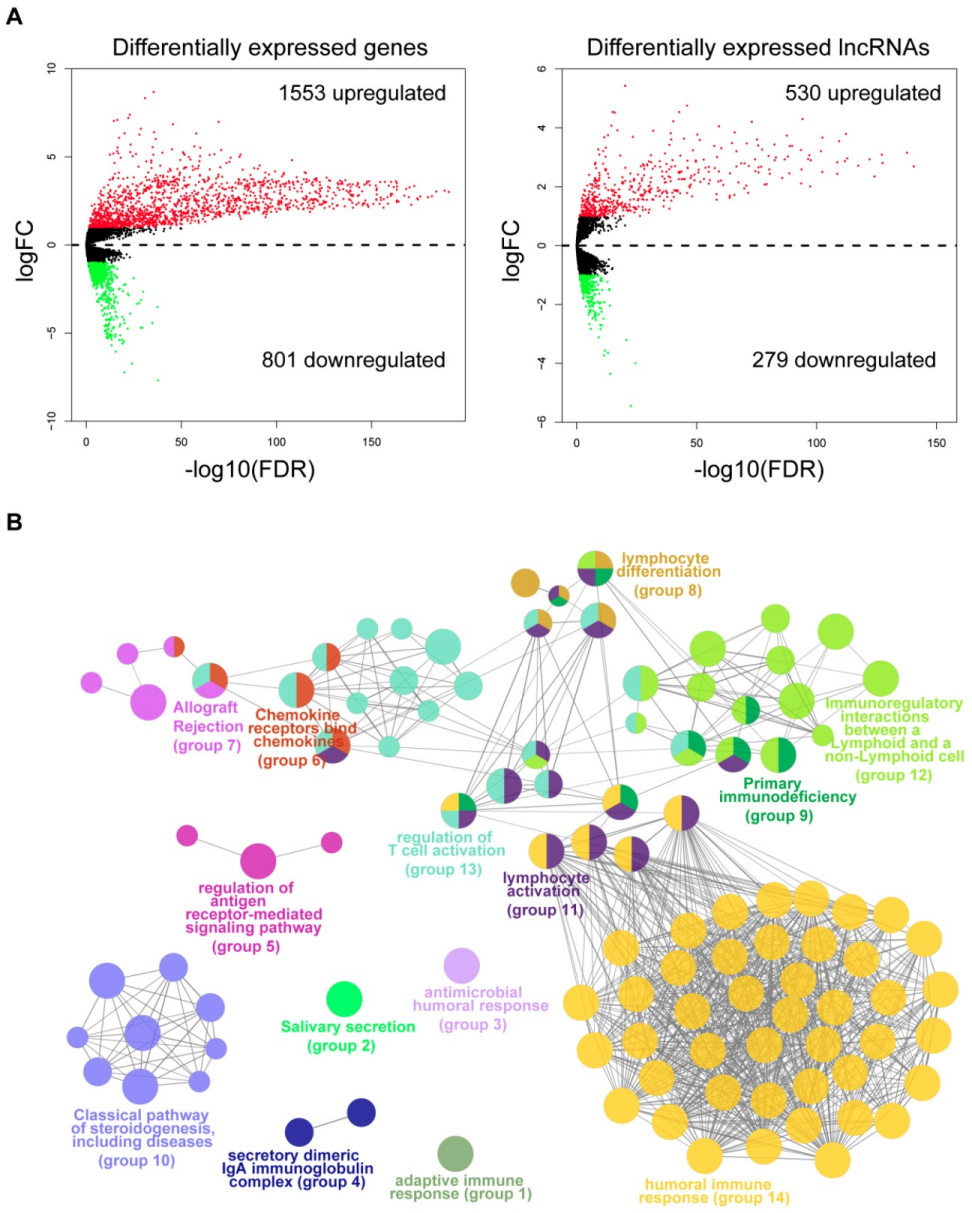
Differential expression analysis and functional annotation. (A) Volcano maps of the differentially expressed mRNAs and lncRNAs. Red points represent the RNAs with a logFC>1 and FDR<0.05. Green points represent RNAs with a logFC<-1 and FDR<0.05. (B) Grouped network of the functional terms enriched in the upregulated genes in the immune_H tumor samples. The nodes represent the enriched terms (adjusted P-value<0.05). The size of the nodes reversely represents the statistical significance of the terms. Functionally related groups partially overlap.

To identify the molecular traits that were associated with immune infiltration, we performed differential expression analysis by comparing tumor samples of the immune_H and immune_L groups from the TCGA cohort. As shown by the volcano maps (Fig. 3A), 1,553 mRNAs and 530 lncRNAs were found to be upregulated in the tissues from the immune_H group, while 801 mRNAs and 279 lncRNAs were downregulated. The top 100 dysregulated RNAs are provided in Table S1-S2. To gain mechanistic insights into the dysregulation between the two groups, we performed a functional analysis of the top upregulated or downregulated genes using ClueGO 22. As a result, 104 functional terms were significantly enriched for the upregulated genes (Table S3). These terms were clustered into 14 groups, the majority of which were closely related to the activation of immune responses (Fig. 3B), such as human immune response (adjusted P=3.78E-70), regulation of T cell activation (adjusted P=2.62E-5), immunoregulatory interactions between a lymphoid and a non-lymphoid cell (adjusted P=1.18E-07) and lymphocyte activation (adjusted P=2.86E-43). In contrast, terms enriched in the downregulated genes were mainly related to skin development and structures (Fig. S6 & Table S4). The results were consistent with the observation that the immune cells were largely infiltrated in those immune_H tumor samples.

### Construction of an immune infiltration-related ceRNA network in CM

**Figure 4:**
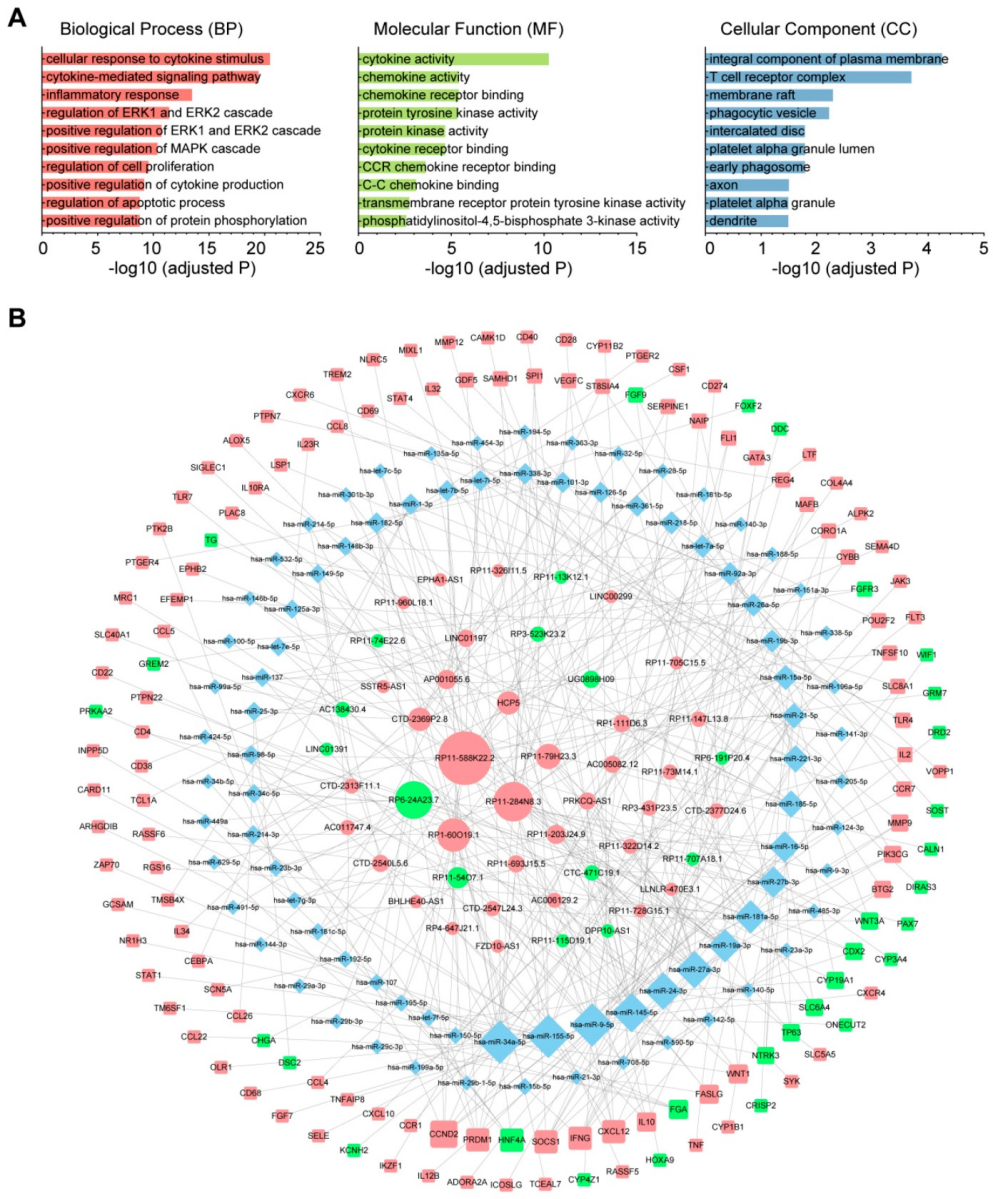
The immune-related ceRNA network. (A) Top 10 GO terms enriched in the ceRNA network. (B) The ceRNA network consisting of 47 lncRNAs, 86 miRNAs and 138 mRNAs was constructed and arranged as a three-layer structure. The ellipses represent lncRNAs, diamonds represent miRNAs and rounded rectangles represent protein-coding genes. The node size is proportional to its degrees. The nodes highlighted in red indicate upregulated expression in the immune_H group, and the nodes labeled green indicate downregulated expression.

To investigate the role of lncRNAs and their interactions with protein-coding genes in the infiltration of immune cells in CM, we constructed a ceRNA network based on the DEmRNAs and DElncRNAs identified above using GDCRNATools 18. The network consisted of 47 lncRNAs, 86 miRNAs and 138 mRNAs with a total of 375 interactions (Table S5). GO analysis confirmed that both immune responses and tumor-intrinsic pathways were significantly enriched in the network (Fig. 4A), such as cellular response to cytokine stimulus, positive regulation of MAPK cascade and protein tyrosine kinase activity. With Cytoscape, the network was arranged into three layers, in which the lncRNAs of the inner layer could modulate the expression of the mRNAs of the outer layer through interactions with the miRNAs of the intermediate layer (Fig. 4B). Notably, 95.3% (82/86) of the miRNAs were denoted to be associated with melanoma by Mammal NcRNA-Disease Repository (MNDR) v2.0 (confidence score>0.4) 28. An examination of the degree distribution of these RNAs revealed a power-law with a slope of -0.747 and 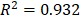, suggesting a typical scale-free structure of biological network. Indeed, the top 10 lncRNAs with highest degrees (hub lncRNAs) controlled more than 78% (108/138) of the genes in this network through competitive binding of 63 miRNAs (Table 1).

**Figure 5:**
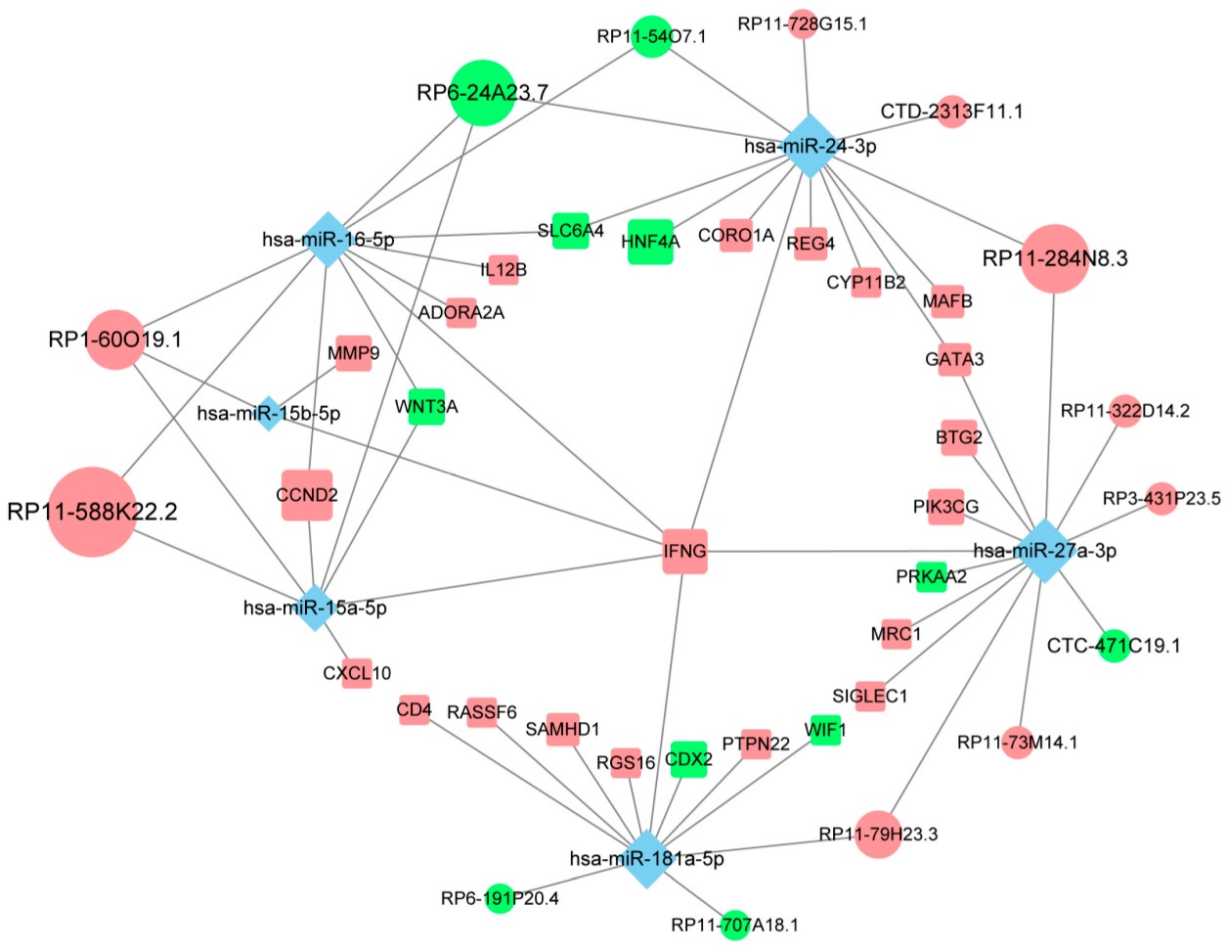
The competing endogenous subnetwork involving *IFNG*. The ellipses represent lncRNAs, diamonds represent miRNAs and rounded rectangles represent protein-coding genes. The node size is proportional to its degrees across the whole network. The nodes highlighted in red indicate upregulated expression in the immune_H group, and the nodes labeled green indicate downregulated expression.

Of all the genes regulated by hub lncRNAs, *IFNG*, whose translational output plays a key role in antitumor immunity 29, was among the most upregulated molecules (logFC=3.74) in the immune_H group while its transcript was under the control of the most miRNA mediators, suggesting that the accumulation of *IFNG* associated with immune infiltration might be positively modulated by those highly competitive lncRNAs, such as RP11-588K22.2, RP11-284N8.3, RP1-60O19.1 and RP11-79H23.3 (Fig. 5). Importantly, most of the lncRNAs remain uncharacterized in CM, which necessitates further focused studies.

### Identification of immune-related prognostic module in CM

**Figure 6:**
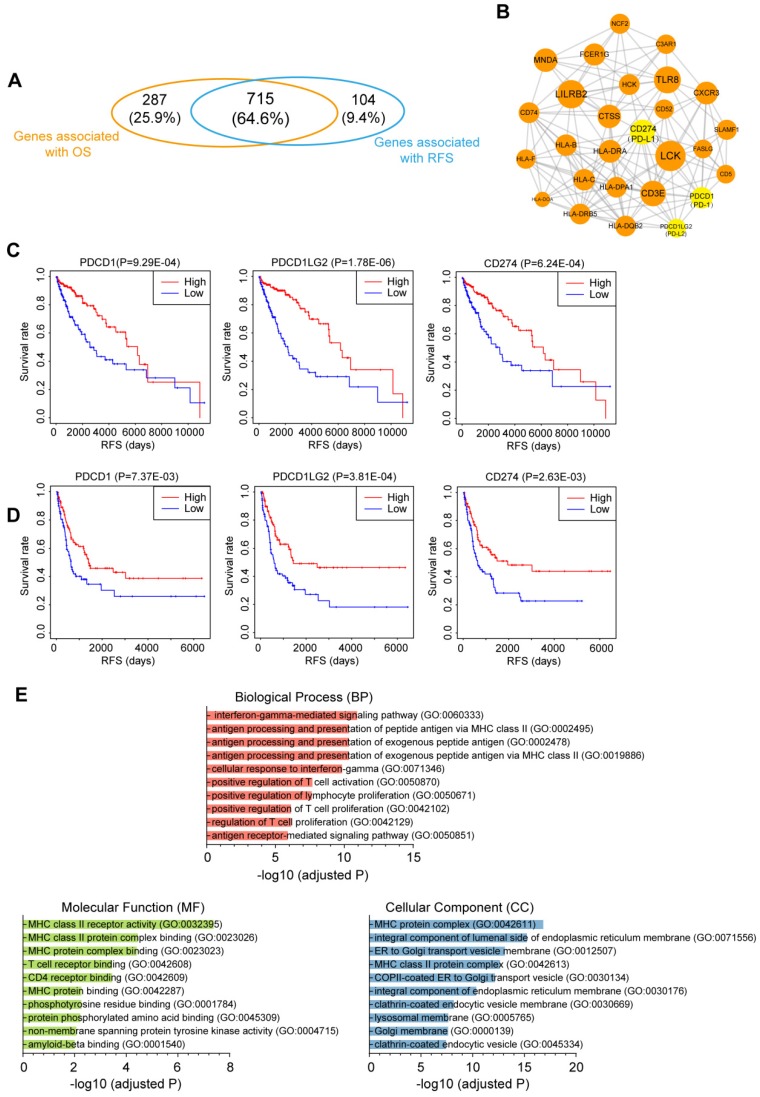
Correlation of the immune-related genes with patient prognosis. (A) Venn diagrams showing the number of overlapped prognostic OS and RFS genes (P<0.01). (B) Selected module from the PPI network constructed using prognostic genes validated by an independent dataset. The key immunomodulators were labeled yellow. (C&D) Kaplan-Meier RFS curves of the immunomodulators in the (C) TCGA and (D) GEO datasets. (E) Top 10 GO terms enriched in the module. OS, overall survival; RFS, relapse-free survival.

To investigate the relationship between immune infiltration and CM patient prognosis at the molecular level, we performed Kaplan-Meier survival analysis of the 2,354 DEmRNAs from the TCGA dataset. As a result, 715 genes were identified to be associated with both OS and RFS (P<0.01; Fig. 6A), among which 109 genes were further tested and validated in the GSE65904 dataset by Kaplan-Meier estimates of RFS (Table S6). All these molecules appeared to be protective, as patients with higher expression levels of these molecules had clearly higher survival rates than those with lower expression levels of these RNAs. To interrogate the interplay among those genes, a PPI network was further constructed (PPI enrichment P-value<1.0E-16). The network comprised 84 nodes and 449 edges (Fig. S7). The hub nodes (the top 15% by degree) included PTPRC, CD86, LCK, LILRB2, CD40LG, TLR8, CD3E, CD274, MNDA, CTSS, HLA-DRA, CXCR3 and FCER1G. These molecules are often central to multiple signaling pathways involved in biological processes like cell proliferation or immune activation, thus influencing more critical functions and making especially attractive drug targets 30. Indeed, ten of the 13 hub genes have targeted drugs in clinic or clinical trials according to Therapeutic target database (Table S6) 31. Furthermore, four subnetwork modules were screened from the whole PPI network and the largest module, which consisted of 27 nodes and 146 edges, is shown in Fig. 6B. Several critical immunomodulators such as PD-1, PD-L1 and PD-L2 were involved in this module, and all the module molecules were positively correlated with patient survival, which indicated that this module played a protective role for CM patients. Figure 6C and 6D illustrates the RFS curves for PD-1, PD-L1 and PD-L2 based on the TCGA and GEO datasets, respectively, with the OS curves displayed in Fig. S8. Functional characterization of this module identified 109 terms from BP, 18 terms from MF and 40 terms from CC that were significantly enriched, the majority of which were closely related to antigen presentation (Fig. 6E). Overall, we discovered a gene module that participates in immune infiltration of CM tissues and leads to favorable outcomes for the patients.

## Discussion

In this study, we performed a comprehensive assessment of the tumor immune microenvironment of CM at three different levels. First, we analyzed the association between immune infiltration and clinicopathological characteristics by considering the TME as a whole (tissue level). Next, we explored the cellular composition of the immune infiltrates in individual tumor tissues to establish potential connections between immune cell subsets and clinical outcomes (cellular level). Finally, we focused on the molecules whose expression profiles were correlated with immune infiltration to discover systems-level regulatory mechanisms and potential prognostic biomarkers (molecular level).

At the tissue level, the correlation between the immune infiltration and clinicopathological characteristics was analyzed. The results suggested that both immune and stromal enrichment status was affected by sex and age in CM. Indeed, the T cell repertoire is generally accepted to decline with increasing age, and the cumulative incidence rate for immune response to melanoma vaccination in older patients has been recently shown to be significantly lower than that in younger patients 32. In addition, males with melanoma generally have worse outcomes than females 33, which can partially be explained by the sex-related differences in the interaction between immune function and tumor biology 34. Our findings that immune infiltration was significantly associated with multiple pathological features, including tumor staging, tumor site, ulceration and tumor thickness, are largely in line with the findings of previous studies 27, 35-37. We also revealed that immune infiltration, not stromal infiltration, was positively correlated with patient survival.

At the cellular level, we found that the five most common immune cells in CM tissues accounted for up to 70% of the immune infiltrates. A comparison study of immune cell fractions showed that tumors with higher immune cell infiltration had significantly higher levels of CD8 T cells, activated memory CD4 T cells and M1 macrophages, while lower levels of M0 and M2 macrophages. In general, CD8 T cells are critical antitumor effector cells, while plasma cells play protective roles in the adaptive immune response. Macrophages exhibit distinct functions based on their activation status. The classically activated M1 macrophages are considered inflammatory (antitumor) while the alternatively activated M2 macrophages are believed to have anti-inflammatory (tumor-promoting) functions. Both types of cells are polarized from uncommitted macrophages (M0) depending on different stimuli. The correlation analysis of these cell types in CM confirmed their functional associations (Fig. 2B). Importantly, these results could largely be validated in independent datasets. Our findings suggested that, despite the heterogeneity in immune cell content across different studies, the immune_H tumors did exhibit enhanced antitumor immunity, which partially explained why patients from this group had an improved clinical outcome. In addition, Kaplan-Meier analysis of the immune cell subsets provided different findings for OS and RFS, which implicated that those immune cells might play a role in preventing the relapse of the disease (Fig. S5B).

At the molecular level, the DEmRNAs and DElncRNAs between immune_H and immune_L groups were identified and used to construct a ceRNA network. As the functional mediators between mRNAs and lncRNAs, 95.3% of the miRNAs in this network were denoted to be melanoma-associated, validating the biological relevance of the network. Based on topological structure, the 10 hub lncRNAs were able to control up to 80% of the mRNAs in this network. Particularly, we found that *IFNG*, which was significantly overexpressed in melanoma tissues with high immune infiltration, was regulated by several of the hub lncRNAs. The controlled expression of *IFNG* is critical for effective antitumor responses. Most previous studies document that the production of its translational output, IFN-γ, is mainly regulated at the transcriptional level by activators or inhibitors 38, while the post-transcriptional regulation is typically inhibitory characterized by miRNA-mediated repression or ARE-mediated decay 39. Our study showed that *IFNG* could also be positively modulated by competing lncRNAs at the post-transcriptional level, as suggested by the upregulation of these lncRNAs along with *IFNG* in the immune_H tumors. Functional analysis suggested that the network is not only involved in the regulation of immune-related pathways, but also regulates tumor-intrinsic pathways in the development of cancer. Recent evidence has shown that oncogenic pathways in tumor cells can be activated to regulate the production of several chemokines and cytokines, which can either decrease the recruitment of immune cells or enhance the recruitment of immunosuppressive cells to tumor sites, contributing to immunoresistance in cancers 40. Indeed, the MAPK pathway in BRAF^V600E^ mutant melanoma cells contributes to comprised function of dendritic cell (DC) in the TME of CM, and the inhibition of the pathway can reverse the suppression of DC function 41. It has also been shown that resistance to BRAF^V600E^ inhibitors (BRAFi) in an autochthonous mouse model of melanoma is associated with restoration of myeloid-derived suppressor cells (MDSC) in the TME, initially reduced by BRAFi treatment, and this process relies upon the reactivation of MAPK pathway 42. Therefore, targeting these oncogenic pathways is a potential strategy for cancer treatment, particularly in combination with immunotherapies such as check point blockers and chimeric antigen receptor-T cell therapy. A deeper understanding of the regulatory relationships between immune response and tumor-intrinsic pathways would lead to new therapeutic strategies that might benefit more CM patients.

Finally, we tested the prognostic relevance of the genes associated with immune infiltration. By Kaplan-Meier analysis and cross validation with GEO dataset, 109 genes were found to be significantly associated with survival of CM patients. The connections between these molecules were interrogated by PPI network analysis, and a highly compact module that conferred prognostic benefit was obtained, which contained several immunotherapeutic targets such as PD-1, PD-L1 and PD-L2. We also noticed that *LCK* was in this module. This gene belongs to the Src family of protein tyrosine kinases and is an integral component of T cell receptor signaling. Previous study identified that high protein expression of *LCK* is strongly correlated with favorable outcomes of CM patients, and tumors from the immune transcriptomic subgroup that correlate with pathological lymphocytic infiltration also express elevated levels of LCK protein 27. Functional analysis suggested that this module participates in antigen presentation.

This study has some limitations. First, the analysis was based on publicly available datasets, in which it was not possible to obtain all relevant information needed for each patient, particularly, the immune-related comorbidities or medication history that might affect the immune microenvironment of that patient. Such patients should ideally be excluded from our study. Second, the results of this study all came from pure bioinformatics analysis. Although we incorporated independent datasets for validation, the results still need to be confirmed by both *in vitro* and *in vivo* experiments. Third, the main purpose of this study was to examine the TME from different levels to screen clinicopathological and molecular features that were associated with immune infiltration in CM and analyzed their potential interconnections. Although we identified some candidate molecules that might impact this process, we did not conduct in-depth analysis on the functions or mechanisms of specific pathways or molecules, which should warrant further focused studies.

## Conclusions

To conclude, we described the immune landscape in detail, suggesting that TME, particularly immune cell infiltration, could have important effects on the pathogenesis of CM and clinical outcomes of the patients. Our study has identified the clinicopathological features, immune cell subsets, differentially expressed RNAs that are associated with immune infiltration. We also constructed an immune-related ceRNA network in CM, in which several of the hub lncRNAs were shown to potentially increase the production of IFN-γ. Finally, we discovered a functional module that contained several validated and potential immunomodulators. Each molecule of the module was illustrated to confer survival advantage for CM patients as supported by OS and RFS rates from different datasets. We believe that our work will advance the understanding of the immune response in the tumor environment and provide valuable resources to explore key molecules and relevant mechanisms related to tumor immunology and immunotherapy in CM.

## Supplementary Material

Supplementary figures and tables.

## Figures and Tables

**Table 1:** 

lncRNAs	miRNAs
RP11-588K22.2	hsa-miR-361-5p, hsa-miR-195-5p, hsa-let-7e-5p, hsa-miR-21-5p, hsa-let-7i-5p, hsa-miR-19a-3p, hsa-miR-19b-3p, hsa-miR-9-5p, hsa-miR-34a-5p, hsa-let-7a-5p, hsa-let-7b-5p, hsa-let-7f-5p, hsa-miR-15a-5p, hsa-miR-126-5p, hsa-miR-454-3p, hsa-miR-16-5p, hsa-miR-182-5p, hsa-miR-98-5p, hsa-miR-135a-5p, hsa-let-7c-5p, hsa-miR-301b-3p
RP11-284N8.3	hsa-miR-24-3p, hsa-miR-361-5p, hsa-miR-126-5p, hsa-miR-19a-3p, hsa-miR-19b-3p, hsa-miR-27a-3p, hsa-miR-23b-3p, hsa-miR-101-3p, hsa-miR-23a-3p, hsa-miR-26a-5p, hsa-miR-27b-3p, hsa-miR-485-3p, hsa-miR-338-3p, hsa-miR-9-3p
RP6-24A23.7	hsa-miR-24-3p, hsa-miR-34a-5p, hsa-miR-34b-5p, hsa-miR-34c-5p, hsa-miR-449a, hsa-miR-629-5p, hsa-miR-15a-5p, hsa-miR-16-5p, hsa-miR-491-5p, hsa-miR-144-3p, hsa-miR-29a-3p, hsa-miR-29b-3p, hsa-miR-29c-3p
RP1-60O19.1	hsa-miR-195-5p, hsa-miR-199a-5p, hsa-miR-150-5p, hsa-miR-218-5p, hsa-miR-15a-5p, hsa-miR-15b-5p, hsa-miR-16-5p, hsa-miR-29b-1-5p, hsa-miR-145-5p, hsa-miR-214-3p, hsa-miR-338-3p
RP11-79H23.3	hsa-miR-361-5p, hsa-miR-181a-5p, hsa-miR-140-3p, hsa-miR-27a-3p, hsa-miR-23b-3p, hsa-miR-181b-5p, hsa-miR-181c-5p
HCP5	hsa-miR-92a-3p, hsa-miR-205-5p, hsa-miR-141-3p, hsa-miR-145-5p, hsa-miR-101-3p, hsa-miR-125a-3p
CTD-2369P2.8	hsa-miR-92a-3p, hsa-miR-25-3p, hsa-miR-32-5p, hsa-miR-363-3p, hsa-miR-194-5p, hsa-miR-107
RP11-54O7.1	hsa-miR-16-5p, hsa-miR-24-3p, hsa-miR-9-5p, hsa-miR-107, hsa-miR-424-5p
AP001055.6	hsa-let-7a-5p, hsa-let-7e-5p, hsa-let-7i-5p, hsa-let-7b-5p
RP11-203J24.9	hsa-let-7g-3p, hsa-miR-19a-3p, hsa-miR-19b-3p, hsa-miR-188-5p

lncRNAs: long noncoding RNAs. miRNAs: microRNAs.
