# Supplementary material for: Comprehensive Analysis of the Tumor Microenvironment in Cutaneous Melanoma associated with Immune Infiltration
Source: J Cancer. 2020 Apr 6;11(13):3858–70. doi: 10.7150/jca.44413 (PMC7171484; doi:10.7150/jca.44413)
Supplement: Supplementary file 1 — Supplementary figures and tables. [file jcav11p3858s1.pdf]

Comprehensive Analysis Of The Tumor Microenvironment In Cutaneous Melanoma Associated With Immune Infiltration

Pan Wang[1,*], Xinyu Zhang[2,*], Nan Sun[1,*], Zhihong Zhao[1], Jie He[1].

[1]Department of Thoracic Surgery, National Cancer Center/National Clinical Research Center for Cancer/Cancer Hospital, Chinese Academy of Medical Sciences and Peking Union Medical College, Beijing 100021, China; [2]Department of body contouring and liposuction center, Plastic Surgery Hospital, Chinese Academy of Medical Sciences and Peking Union Medical College, Beijing 100144, China. *These authors contributed equally to this work.

Correspondence to: Jie He, E-mail: prof.jiehe@gmail.com. National Cancer Center/National Clinical Research Center for Cancer/Cancer Hospital, Chinese Academy of Medical Sciences and Peking Union Medical College, Beijing 100021, China. Tel: 86-10-87788863. Fax numbers: +86-10-67713359.

Table S1. Top 100 differentially expressed genes between tumors with higher immune scores and those with lower immune scores.

gene symbol	log2FC	FDR	gene symbol	log2FC	FDR
CYP11B1	8.6784634	3.92E-36	SCGB1A1	-7.685453	1.52E-38
STATH	8.3344156	1.19E-31	IZUMO3	-7.232034	7.65E-21
PTH	7.3971689	1.34E-23	TBC1D21	-6.722844	8.04E-25
AMY1B	7.216505	7.64E-23	KRTAP9-7	-6.050419	3.52E-16
MYF5	7.0954607	4.83E-17	KRTAP3-1	-5.689618	4.57E-14
SMR3B	7.0448764	2.94E-15	KRT83	-5.592111	8.66E-20
CYP17A1	6.9883843	2.42E-70	KRT33B	-5.47886	2.12E-20
PRH2	6.8527637	2.62E-46	KRT9	-5.395645	3.47E-18
GPR101	6.5964381	2.09E-29	KRTAP1-3	-5.370988	4.73E-14
HSD3B2	6.4033855	2.26E-36	LALBA	-5.370131	1.29E-19
MC2R	6.3369643	6.99E-21	KRTAP4-9	-5.298941	3.93E-12
IGKV1D-17	6.3312213	1.42E-58	KRTAP4-7	-5.249731	1.99E-11
BPIFA2	6.2611682	1.18E-28	KRTAP2-1	-5.24692	9.50E-14
PRB4	6.0788263	5.20E-18	KRTAP1-1	-5.206452	6.33E-16
IGKV2D-28	6.0224548	2.98E-37	KRTAP10-7	-5.204526	3.90E-12
SPANXB2	5.7007386	4.56E-24	KRTAP4-6	-5.193143	6.14E-12
IGKV2D-24	5.5928723	1.34E-57	KRTAP10-5	-5.172852	2.78E-13
PRB3	5.5526067	1.12E-35	KRTAP4-8	-5.156828	2.51E-12
OR8K5	5.5336801	3.68E-17	KRTAP10-9	-5.120953	2.93E-14
CR2	5.5194701	1.09E-60	KRT33A	-5.120226	1.34E-18
CYP21A2	5.4285799	8.09E-46	KRTAP10-1	-5.081823	5.53E-16
STAR	5.3874627	1.15E-71	KRTAP3-3	-5.065946	2.10E-13
PRR27	5.3258834	6.54E-17	KRTAP4-4	-5.040963	2.00E-11
PRB2	5.2723347	7.52E-24	KRTAP2-4	-5.013304	3.21E-12
IGHV2-70	5.2638971	1.37E-51	KRTAP9-3	-4.896354	1.77E-10
IGKV2D-30	5.2431461	9.35E-41	KRTAP4-3	-4.88627	2.07E-11

IGLV9-49	5.2029196	7.77E-52	KRTAP10-3	-4.875783	8.11E-14
REG1A	5.2011527	1.50E-17	KRTAP9-9	-4.821685	7.22E-11
HTN3	5.1891161	5.16E-15	AADACL3	-4.767987	3.64E-12
CSN3	5.1669594	7.10E-27	AWAT1	-4.742701	1.72E-22
PRH1	5.1593225	3.95E-68	KRTAP10-10	-4.742325	1.72E-12
FCER2	5.1320324	8.46E-82	KRTAP10-8	-4.71501	1.06E-14
REG1B	5.1297695	4.09E-13	KRTAP3-2	-4.714543	3.79E-15
IGHV3-73	5.1278712	2.00E-52	OSTN	-4.700519	3.31E-30
IGKV1D-33	5.125716	5.68E-36	ST8SIA3	-4.688022	1.69E-29
IGKV2-24	5.1131972	1.34E-65	KRTAP9-4	-4.650434	3.43E-15
LRRC38	5.0442645	4.16E-25	KRTAP9-6	-4.619802	2.35E-18
LPO	5.036557	1.39E-60	KRTAP9-8	-4.601118	1.01E-11
MS4A1	5.0012389	2.66E-81	KRTAP4-2	-4.597655	2.51E-10
FCAMR	4.99594	1.10E-54	KRTAP4-5	-4.580925	7.86E-11
IGLV1-51	4.9540555	1.21E-76	KRTAP2-2	-4.556507	1.87E-12
SPANXC	4.9480993	7.91E-19	KRTAP8-1	-4.533326	1.70E-11
IGKV1D-16	4.8533495	1.82E-55	KRTAP4-11	-4.51617	2.84E-11
IGKV1D-43	4.8254076	4.06E-50	H2BFWT	-4.495647	1.37E-18
PLA2G2D	4.8161235	5.85E-109	KRTAP11-1	-4.441994	1.22E-12
IGKV2-40	4.7996369	6.78E-35	KRTAP1-5	-4.436329	8.33E-21
IGHV3-64	4.7592057	3.15E-60	MPPED1	-4.418423	1.49E-35
IGKV2-28	4.7422667	2.72E-32	KRTAP10-11	-4.273631	4.16E-13
IGHM	4.7190972	5.17E-76	KRTAP17-1	-4.261905	1.79E-11
IGHD3-16	4.6786288	7.60E-35	KRTAP5-5	-4.253494	3.03E-13
TCL1A	4.6611426	1.85E-74	AWAT2	-4.240798	1.88E-20
SPANXD	4.6580061	1.42E-15	KRTAP10-4	-4.238978	4.01E-11
CNR2	4.5164502	6.83E-92	SCRT2	-4.234487	3.81E-15
IGHD2-2	4.5023175	1.29E-39	KRT85	-4.199503	2.32E-12
FCRL1	4.497572	7.50E-60	KRTAP10-2	-4.193041	1.49E-13

IGKV1-16	4.4664626	1.09E-56	C20orf85	-4.128713	5.72E-17
SBK2	4.4473911	3.39E-28	OR2V1	-4.078444	4.27E-14
CXCR5	4.4106071	3.44E-71	CYP4F8	-4.076273	9.81E-20
IGLV1-36	4.4026673	1.71E-52	KRTAP4-1	-4.028033	2.54E-16
IGLV1-47	4.4016726	2.38E-53	KRT35	-4.011	9.44E-10
IGKV2D-40	4.3686625	4.94E-35	KRTAP12-2	-3.945415	1.01E-11
TNFRSF13B	4.3598921	5.09E-89	KRTAP12-1	-3.892213	1.90E-10
IGKV1D-8	4.3489472	2.08E-59	MUC5AC	-3.890291	8.05E-24
ZG16B	4.3379807	4.09E-52	KRTAP10-12	-3.870649	3.53E-13
BANK1	4.3291873	5.97E-91	TAC1	-3.797457	3.52E-16
IGHD	4.3274719	4.75E-58	LCE3C	-3.711313	4.50E-09
IGHD3-3	4.3208201	9.01E-45	GCG	-3.693766	2.75E-09
IGHV3-7	4.3186332	1.37E-48	KRTAP10-6	-3.60921	4.00E-12
PAX5	4.2884488	1.13E-65	KRTAP7-1	-3.59803	1.27E-15
CD19	4.2786979	5.06E-83	KRTAP4-12	-3.594669	3.77E-10
IGHV1-18	4.2480549	7.58E-57	MS4A15	-3.588427	5.42E-14
FCRL4	4.2207046	4.06E-45	KRTAP5-3	-3.55161	1.13E-10
FAM129C	4.1783487	5.40E-100	SEZ6	-3.517124	3.41E-38
IGKV1-39	4.1694751	8.47E-44	LY6G6D	-3.479255	3.32E-12
STAP1	4.1283166	1.22E-115	MYOC	-3.463777	4.77E-15
IGKV1D-12	4.1215837	2.80E-33	FOXI3	-3.457972	3.19E-14
TRBV12-4	4.1158632	7.02E-95	FABP9	-3.425196	1.02E-08
CCL21	4.1140341	6.62E-50	PM20D1	-3.341206	1.83E-27
TACR3	4.1044771	6.62E-18	KRTAP5-4	-3.338049	8.00E-13
TIMD4	4.0861462	1.27E-104	ELSPBP1	-3.327486	4.21E-13
IGKV3D-7	4.0833611	5.54E-50	KCNMB2	-3.289611	1.37E-28
FCRL3	4.0820408	3.89E-116	KRTAP5-7	-3.28035	2.70E-17
AMY1A	4.040253	8.65E-16	KERA	-3.270271	1.81E-15
TRBV10-3	4.0057746	8.37E-97	GNAT3	-3.239131	1.48E-11

FCRL2	3.9973028	3.11E-70	KRT25	-3.237728	3.13E-07
TRBV6-6	3.9970233	2.17E-104	BSND	-3.220848	4.53E-11
CD79A	3.9784361	4.39E-87	KRT34	-3.220184	3.15E-12
IRG1	3.9708891	1.82E-60	SNTN	-3.219734	1.51E-15
IGHV3-48	3.9705983	9.81E-50	KRT28	-3.214331	6.88E-07
IGLV8-61	3.9684999	2.17E-37	CSN2	-3.202639	3.63E-07
IGKV1-12	3.9409508	3.85E-41	KRT71	-3.189447	2.04E-06
CLEC4M	3.9369895	1.26E-39	GABRA4	-3.160981	1.41E-15
CTD-2207O23.3	3.9329582	1.70E-60	HMX1	-3.133451	2.38E-13
TLR10	3.8938976	1.29E-103	KRT26	-3.117387	1.24E-09
IGLV7-43	3.8810879	2.16E-43	TG	-3.110697	1.58E-29
IGHV3OR16-8	3.8800287	5.71E-47	KRTAP9-2	-3.088047	6.43E-14
BLK	3.8745257	3.43E-72	TNMD	-3.083902	2.78E-12
TRBV10-2	3.8679844	5.13E-70	CTXN3	-3.07716	1.30E-11
IGKV1D-13	3.8559384	5.29E-32	KRTAP12-3	-3.071098	1.39E-10
AICDA	3.851646	4.13E-55	XXbac-BPG32J3.19	-3.070139	3.58E-11

log2FC, log values of expression fold changes; FDR, false discovery rate.

Table S2. The top 100 differentially expressed lncRNAs between tumors with higher immune scores and those with lower immune scores.

lncRNA symbol	log2FC	FDR	lncRNA symbol	log2FC	FDR
LINC00824	6.0425233	1.54E-54	CTD-2587M23.1	-5.43742	2.56E-23
LINC00207	5.42915	6.71E-21	CTD-3138F19.1	-4.353638	8.92E-15
RP11-164H13.1	4.7526759	9.46E-47	FAM41C	-3.99078	3.19E-25
LINC00347	4.5382915	9.69E-16	RP11-2L8.2	-3.725457	4.07E-12
RP11-297B17.3	4.5348328	1.86E-43	GPC5-AS1	-3.639711	8.55E-14

RP11-458K10.1	4.5189574	1.18E-16	CTC-261N6.1	-3.562658	1.67E-12
LINC01215	4.2998269	6.92E-95	C7orf71	-3.20058	1.77E-21
RP11-203B7.2	4.2282237	4.94E-41	RP11-98O2.1	-3.110219	6.03E-10
LINC00402	4.2051945	9.53E-74	CTD-2296D1.1	-2.834117	1.15E-10
LINC00967	4.0721903	3.17E-15	RP11-521M14.1	-2.831573	1.44E-06
RP11-731F5.2	4.0537616	4.39E-60	GRM5-AS1	-2.704605	3.15E-11
RP11-231I13.2	3.9175564	9.87E-18	RP11-442J17.3	-2.695389	1.52E-10
RP11-132N15.3	3.9012577	1.13E-32	LINC01582	-2.66352	5.09E-06
KIAA0125	3.8445763	1.29E-66	CTD-2544M6.1	-2.518635	8.66E-09
RP11-61O1.2	3.8171757	5.30E-40	RP11-561I11.3	-2.47946	6.96E-12
LINC00861	3.7926739	5.39E-113	RP5-912I13.1	-2.468182	2.13E-07
AL122127.25	3.7702418	4.94E-51	RMST	-2.43	2.23E-08
RP11-217E22.5	3.7549934	9.39E-14	CTC-565M22.1	-2.401472	3.67E-08
RP11-291B21.2	3.7372375	2.53E-88	RP11-10O22.1	-2.354136	5.66E-09
CTA-833B7.2	3.6940383	3.37E-24	RP11-398B16.2	-2.347969	1.01E-05
MIR202HG	3.6808427	2.93E-12	RP11-739N10.1	-2.333428	5.80E-10

TRBV11-2	3.6681836	3.43E-103	LINC01284	-2.326098	2.59E-08
RP5-887A10.1	3.6355309	1.79E-45	AC009878.2	-2.267695	5.93E-06
GTSCR1	3.6079314	3.63E-40	AC073834.3	-2.260544	1.97E-06
RP11-542M13.3	3.6047471	6.87E-76	CTC-471C19.1	-2.259944	1.65E-08
RP11-768B22.2	3.5877125	4.82E-52	RP11-802F5.1	-2.244756	3.93E-11
AC079767.4	3.5475308	3.75E-110	RP11-255H23.4	-2.213763	1.12E-07
SIRPG-AS1	3.4819636	1.67E-65	AC006552.1	-2.19562	3.37E-06
RP11-109E24.1	3.469815	1.19E-71	LINC01497	-2.188029	1.36E-06
CTD-2313F11.1	3.4330593	3.48E-83	RP11-179K3.2	-2.178589	1.43E-06
RP11-138I18.2	3.4216876	2.18E-39	LINC01429	-2.134588	5.42E-11
RP11-10J5.1	3.4004203	1.96E-76	RP3-449H6.1	-2.107487	2.87E-10
LINC01501	3.3855159	4.36E-58	AP000479.1	-2.074436	3.75E-06
RP11-1399P15.1	3.381421	1.42E-90	RP11-167B3.1	-2.063788	0.0001198
RP11-493L12.5	3.3808627	1.44E-89	RP11-572C21.1	-2.057292	1.52E-07
RP11-285E18.2	3.3289432	6.89E-13	CTD-2626G11.2	-2.056609	2.78E-07

RP11-428G5.5	3.3076523	4.64E-61	AP000487.4	-2.034943	1.42E-14
RP11-202G18.1	3.2647583	4.40E-78	RP11-110A12.2	-2.028319	5.61E-07
CTD-2161F6.2	3.2641249	9.27E-10	CTD-2147F2.1	-1.983555	6.89E-06
AC009133.17	3.248021	2.88E-94	RP11-64B16.4	-1.981068	1.28E-07
IFNG-AS1	3.2395872	3.13E-49	RP4-539M6.14	-1.966598	3.21E-10
AC064834.1	3.2135164	9.00E-12	RP11-159N11.3	-1.94682	1.78E-08
AC092580.4	3.2117897	1.27E-102	LINC00844	-1.935183	6.51E-08
CTD-2506J14.1	3.2054628	2.43E-67	LINC01077	-1.924672	6.46E-11
ITGB2-AS1	3.1521766	1.96E-138	RP4-806M20.5	-1.92272	5.35E-06
AC097713.4	3.1343848	1.46E-37	KIAA0087	-1.920986	2.59E-12
RP13-379L11.1	3.094663	3.11E-10	RP11-13K12.2	-1.908546	1.20E-09
USP30-AS1	3.0889766	5.62E-128	AC012499.1	-1.906787	5.93E-07
AC104820.2	3.0703558	9.62E-98	RP5-827O9.1	-1.8958	4.68E-05
RP11-736N17.8	3.0689856	1.13E-31	LINC00508	-1.895316	4.69E-05
GLIS3-AS1	3.0592385	6.42E-19	AL121578.2	-1.891614	2.25E-06
RP11-693J15.5	3.0561287	5.59E-44	RP11-78H24.1	-1.86919	1.45E-05

RP11-18H21.1	3.0467764	9.09E-81	RP4-668E10.4	-1.856431	7.14E-07
RP11-960L18.1	3.0290665	9.06E-89	LINC00162	-1.830813	3.76E-07
RP11-538I12.3	3.0185398	9.55E-13	AC010091.1	-1.794599	0.0013734
RP11-598F7.3	3.0084454	2.80E-59	LLNLR-249E10.1	-1.769859	1.81E-05
AC083949.1	2.9973141	8.33E-84	LINC01468	-1.76629	1.61E-07
PCED1B-AS1	2.9773241	4.48E-183	LINC00890	-1.758259	6.86E-06
AP003774.1	2.9616358	1.62E-113	RP4-718N17.2	-1.755449	0.0013658
RP5-1171I10.5	2.9408222	2.90E-97	LINC00165	-1.749398	8.69E-07
AC006129.2	2.9315966	3.85E-75	RP11-457P14.5	-1.737335	1.25E-06
RP11-327F22.2	2.930169	7.19E-123	RP11-527N12.2	-1.730556	8.37E-05
RP13-580F15.2	2.9290395	1.74E-87	AC053503.11	-1.729321	2.45E-07
LINC01055	2.922782	1.10E-53	RP11-563P16.1	-1.669806	2.00E-08
LINC00544	2.903661	1.10E-40	RP11-789C1.1	-1.663605	0.0011278
AC023590.1	2.9013294	2.69E-83	CXADRP3	-1.653599	1.46E-05
LINC00996	2.8989409	2.60E-98	UG0898H09	-1.649409	3.95E-08

RP11-553L6.2	2.8921325	2.30E-50	RP5-963E22.4	-1.632244	4.15E-10
AC013264.2	2.8840551	6.31E-47	AC109309.4	-1.625932	2.70E-05
RP11-161M6.3	2.8686532	2.20E-35	RP11-561I11.4	-1.613948	6.16E-05
RP11-455F5.5	2.8684975	1.49E-112	AC105402.4	-1.611719	4.54E-06
PP12613	2.859133	7.03E-31	LINC00682	-1.604239	2.10E-06
AC104699.1	2.8490865	1.74E-50	RP11-510C10.2	-1.593931	0.0032335
PIK3CD-AS1	2.8307746	2.28E-109	AC092570.2	-1.581804	0.0005093
RP1-148H17.1	2.8295005	7.73E-32	NRG1-IT1	-1.58116	6.59E-05
RP11-680N20.1	2.8143007	1.56E-08	AC008060.7	-1.578499	0.0001946
AC133644.2	2.8067783	2.83E-128	RP11-244B22.11	-1.569566	0.0009107
RP11-16E12.2	2.7928566	2.47E-69	RP11-557H15.5	-1.566347	0.0003457
RP11-284N8.3	2.7922973	9.93E-71	RP11-465B22.8	-1.562254	1.31E-05
RP11-2N1.2	2.7812079	9.19E-12	FAM83A-AS1	-1.559199	1.72E-05
RP11-342D11.3	2.7712936	3.48E-38	AC110781.3	-1.541314	3.11E-07
CTD-2066L21.2	2.7605926	2.35E-08	LINC00948	-1.538889	2.40E-07

AC093642.4	2.7601569	2.37E-28	LINC00454	-1.532212	0.003091
RP11-44K6.4	2.7576131	4.71E-30	DPP10-AS1	-1.529026	0.0009577
AC096579.13	2.7266026	3.27E-30	RP5-1056H1.2	-1.528722	7.82E-05
RP4-647J21.1	2.7136046	4.08E-118	LINC01391	-1.517676	1.86E-07
RP11-554I8.1	2.7122654	8.78E-29	RP11-89N17.4	-1.515822	2.29E-14
RP11-1094M14.5	2.7052034	1.73E-72	AC012506.1	-1.515551	0.0004544
RP11-664D1.1	2.7007571	9.68E-42	RP11-510C10.3	-1.51246	0.0057503
RP11-73G16.2	2.698995	1.99E-33	RP11-706C16.7	-1.512231	8.18E-06
RP11-44K6.2	2.6941669	1.62E-27	RP4-566L20.1	-1.511173	3.88E-08
RP5-1091N2.9	2.6902779	2.80E-141	AC091729.8	-1.506489	2.08E-11
RP13-297E16.4	2.6902695	2.30E-67	LINC01297	-1.503101	2.00E-05
LINC00582	2.6783572	3.20E-40	CTD-2072I24.1	-1.502976	6.61E-07
UNQ6494	2.6756408	1.17E-77	RP11-617I14.1	-1.501285	1.18E-07
RP11-23P13.6	2.6732917	7.77E-56	AC060834.3	-1.500878	0.0009693
RP11-705C15.5	2.6732176	1.66E-35	RP6-191P20.4	-1.496962	3.12E-07

RP11-493L12.4	2.6555071	1.38E-76	DLX6-AS1	-1.494069	1.34E-09
RP11-212I21.5	2.6467785	6.31E-47	RP11-520D19.2	-1.491081	1.02E-08
RP13-452N2.1	2.6437119	1.80E-54	RP3-410C9.2	-1.482437	0.0023663

lncRNA, long non-coding RNA.

Table S3. Detailed enrichment results of the top upregulated genes in tumors with higher immune scores.

Groups	GOID	GOTerm	Adjusted P-Value
Group1	GO:0002250	adaptive immune response	1.20E-174
Group2	KEGG:04970	Salivary secretion	1.03E-04
Group3	GO:0019730	antimicrobial humoral response	1.78E-04
Group4	GO:0071748	monomeric IgA immunoglobulin complex	2.06E-03
Group4	GO:0071752	secretory dimeric IgA immunoglobulin complex	5.21E-04
Group5	GO:0050854	regulation of antigen receptor-mediated signaling pathway	1.03E-04
Group5	GO:0050857	positive regulation of antigen receptor-mediated signaling pathway	9.02E-03
Group5	GO:0050855	regulation of B cell receptor signaling pathway	2.30E-02
Group6	KEGG:04060	Cytokine-cytokine receptor interaction	1.87E-04
Group6	R-HSA:380108	Chemokine receptors bind chemokines	3.69E-06
Group6	WP:2328	Allograft Rejection	1.02E-05
Group6	GO:1990869	cellular response to chemokine	1.93E-03
Group7	KEGG:05330	Allograft rejection	3.44E-02

Group7	KEGG:04650	Natural killer cell mediated cytotoxicity	1.89E-02
Group7	GO:0031343	positive regulation of cell killing	2.23E-04
Group7	GO:0042267	natural killer cell mediated cytotoxicity	1.53E-02
Group8	GO:0022407	regulation of cell-cell adhesion	3.30E-03
Group8	GO:0046651	lymphocyte proliferation	4.26E-02
Group8	GO:0042100	B cell proliferation	1.67E-03
Group8	GO:0030098	lymphocyte differentiation	8.43E-07
Group8	GO:0050870	positive regulation of T cell activation	2.46E-04
Group9	KEGG:04660	T cell receptor signaling pathway	3.02E-04
Group9	KEGG:05340	Primary immunodeficiency	4.06E-10
Group9	GO:0042110	T cell activation	7.25E-10
Group9	GO:0045058	T cell selection	4.34E-03
Group9	GO:0030217	T cell differentiation	2.36E-04
Group10	KEGG:00140	Steroid hormone biosynthesis	3.66E-02
Group10	R-HSA:193993	Mineralocorticoid biosynthesis	1.01E-02
Group10	R-HSA:194002	Glucocorticoid biosynthesis	1.21E-05
Group10	R-HSA:196071	Metabolism of steroid hormones	1.34E-03
Group10	R-HSA:5579029	Metabolic disorders of biological oxidation enzymes	2.30E-02
Group10	WP:237	Glucocorticoid and Mineralcorticoid Metabolism	4.84E-04
Group10	WP:4523	Classical pathway of steroidogenesis, including diseases	9.84E-06
Group10	GO:0008211	glucocorticoid metabolic process	7.58E-04
Group10	GO:0006705	mineralocorticoid biosynthetic process	4.68E-03
Group11	GO:0045321	leukocyte activation	5.68E-28
Group11	GO:0046649	lymphocyte activation	2.86E-43
Group11	GO:0002694	regulation of leukocyte activation	1.63E-34

Group11	GO:0007159	leukocyte cell-cell adhesion	5.59E-05
Group11	GO:0022409	positive regulation of cell-cell adhesion	1.33E-03
Group11	GO:0051249	regulation of lymphocyte activation	2.54E-37
Group11	GO:0031295	T cell costimulation	2.61E-03
Group12	KEGG:04640	Hematopoietic cell lineage	2.19E-06
Group12	R-HSA:198933	Immunoregulatory interactions between a Lymphoid and a non-Lymphoid cell	1.18E-07
Group12	R-HSA:202427	Phosphorylation of CD3 and TCR zeta chains	2.17E-03
Group12	R-HSA:202430	Translocation of ZAP-70 to Immunological synapse	9.91E-04
Group12	R-HSA:202433	Generation of second messenger molecules	1.72E-02
Group12	R-HSA:389948	PD-1 signaling	1.14E-04
Group12	WP:3863	T-Cell antigen Receptor (TCR) pathway during Staphylococcus aureus infection	4.39E-02
Group12	GO:0042101	T cell receptor complex	7.36E-06
Group12	GO:0042105	alpha-beta T cell receptor complex	4.84E-04
Group12	GO:0050852	T cell receptor signaling pathway	2.64E-03
Group13	GO:0050863	regulation of T cell activation	2.62E-05
Group13	R-HSA:373076	Class A/1 (Rhodopsin-like receptors)	2.88E-03
Group13	R-HSA:375276	Peptide ligand-binding receptors	9.89E-05
Group13	WP:24	Peptide GPCRs	1.67E-02
Group13	GO:0002407	dendritic cell chemotaxis	1.07E-02
Group13	GO:0002408	myeloid dendritic cell chemotaxis	2.77E-02

Group13	GO:2000547	regulation of dendritic cell dendrite assembly	2.06E-03
Group13	GO:2000510	positive regulation of dendritic cell chemotaxis	4.11E-02
Group13	GO:2000669	negative regulation of dendritic cell apoptotic process	2.77E-02
Group14	GO:0002682	regulation of immune system process	6.00E-52
Group14	GO:0009986	cell surface	6.11E-24
Group14	GO:0098552	side of membrane	2.54E-27
Group14	GO:0002443	leukocyte mediated immunity	6.95E-35
Group14	GO:0002684	positive regulation of immune system process	9.87E-54
Group14	GO:0006959	humoral immune response	3.78E-70
Group14	GO:0042571	immunoglobulin complex, circulating	5.56E-46
Group14	GO:0051707	response to other organism	4.66E-21
Group14	GO:0061024	membrane organization	2.82E-10
Group14	GO:0002697	regulation of immune effector process	5.24E-29
Group14	GO:0045087	innate immune response	2.03E-17
Group14	GO:0050776	regulation of immune response	2.02E-53
Group14	GO:0050900	leukocyte migration	1.10E-32
Group14	GO:0002449	lymphocyte mediated immunity	1.27E-63
Group14	GO:0002460	adaptive immune response based on somatic recombination of immune receptors built from immunoglobulin superfamily domains	1.09E-59
Group14	GO:0009617	response to bacterium	2.54E-25
Group14	GO:0050778	positive regulation of immune response	1.31E-54
Group14	GO:0098542	defense response to other organism	1.89E-19

Group14	GO:0002253	activation of immune response	1.53E-53
Group14	GO:0002696	positive regulation of leukocyte activation	7.66E-33
Group14	GO:0002920	regulation of humoral immune response	1.25E-50
Group14	GO:0006910	phagocytosis, recognition	5.50E-41
Group14	GO:0009897	external side of plasma membrane	6.14E-34
Group14	GO:0042113	B cell activation	3.52E-37
Group14	GO:0002757	immune response-activating signal transduction	1.64E-55
Group14	GO:0002768	immune response-regulating cell surface receptor signaling pathway	5.06E-63
Group14	GO:0006897	endocytosis	9.76E-33
Group14	GO:0006909	phagocytosis	3.40E-51
Group14	GO:0006956	complement activation	1.51E-69
Group14	GO:0019724	B cell mediated immunity	1.85E-66
Group14	GO:0042742	defense response to bacterium	1.09E-26
Group14	GO:0002455	humoral immune response mediated by circulating immunoglobulin	7.86E-70
Group14	GO:0051251	positive regulation of lymphocyte activation	3.11E-34
Group14	GO:0006898	receptor-mediated endocytosis	4.76E-24
Group14	GO:0006911	phagocytosis, engulfment	1.43E-37
Group14	GO:0030449	regulation of complement activation	3.56E-48
Group14	GO:0038093	Fc receptor signaling pathway	1.66E-31
Group14	GO:0050864	regulation of B cell activation	1.40E-37
Group14	GO:0050871	positive regulation of B cell activation	1.29E-35
Group14	GO:0038095	Fc-epsilon receptor signaling pathway	2.80E-34

Group14	GO:0050851	antigen receptor-mediated signaling pathway	2.24E-41
Group14	GO:0038096	Fc-gamma receptor signaling pathway involved in phagocytosis	1.15E-40
Group14	GO:0050853	B cell receptor signaling pathway	4.01E-44

Table S4. Detailed enrichment results of the top downregulated genes in tumors with higher immune scores.

Groups	GOID	GOTerm	Adjusted P-Value
Group1	GO:0045104	intermediate filament cytoskeleton organization	2.18E-02
Group2	GO:0046883	regulation of hormone secretion	4.26E-02
Group3	WP:272	Blood Clotting Cascade	3.23E-02
Group4	WP:2840	Hair Follicle Development: Cytodifferentiation (Part 3 of 3)	3.05E-02
Group5	WP:2877	Vitamin D Receptor Pathway	9.79E-06
Group6	GO:0042633	hair cycle	1.86E-07
Group7	R-HSA:8964058	HDL remodeling	3.51E-02
Group7	GO:0019433	triglyceride catabolic process	3.97E-02
Group8	KEGG:00830	Retinol metabolism	4.38E-02
Group8	R-HSA:211859	Biological oxidations	4.34E-02
Group8	R-HSA:211981	Xenobiotics	4.51E-02
Group9	KEGG:04915	Estrogen signaling pathway	1.46E-06
Group9	R-HSA:6809371	Formation of the cornified envelope	2.30E-40
Group9	GO:0070268	cornification	2.35E-38
Group10	R-HSA:1266738	Developmental Biology	8.77E-49
Group10	R-HSA:6805567	Keratinization	5.09E-113
Group10	GO:0009888	tissue development	7.02E-30
Group10	GO:0099512	supramolecular fiber	2.21E-34

Group10	GO:0008544	epidermis development	9.72E-81
Group10	GO:0060429	epithelium development	4.85E-44
Group10	GO:0030855	epithelial cell differentiation	1.01E-59
Group10	GO:0043588	skin development	7.55E-87
Group10	GO:0044430	cytoskeletal part	6.66E-20
Group10	GO:0031424	keratinization	9.22E-117
Group10	GO:0030216	keratinocyte differentiation	3.04E-99
Group10	GO:0045111	intermediate filament cytoskeleton	8.93E-81
Group10	GO:0099513	polymeric cytoskeletal fiber	2.65E-42
Group10	GO:0045095	keratin filament	6.22E-78

Table S5. The immune infiltration-related ceRNA network of CM.

ceRNA symbol	biotype†	log2FC‡	miRNA id	Oncomir confidence score§
CTD-2313F11.1	processed_transcript	3.4330593	hsa-miR-24-3p	0.4752
CTD-2313F11.1	processed_transcript	3.4330593	hsa-miR-149-5p	0.8808
RP11-693J15.5	lincRNA	3.0561287	hsa-miR-155-5p	0.7606
RP11-693J15.5	lincRNA	3.0561287	hsa-miR-34a-5p	0.8939
RP11-693J15.5	lincRNA	3.0561287	hsa-miR-124-3p	0.8808
RP11-960L18.1	lincRNA	3.0290665	hsa-miR-148b-3p	0.9578
AC006129.2	lincRNA	2.9315966	hsa-miR-145-5p	0.4752
AC006129.2	lincRNA	2.9315966	hsa-miR-140-5p	0.8939
RP11-284N8.3	lincRNA	2.7922973	hsa-miR-24-3p	0.4752
RP11-284N8.3	lincRNA	2.7922973	hsa-miR-361-5p	0.4752
RP11-284N8.3	lincRNA	2.7922973	hsa-miR-126-5p	0.4752
RP11-284N8.3	lincRNA	2.7922973	hsa-miR-19a-3p	0.8808
RP11-284N8.3	lincRNA	2.7922973	hsa-miR-19b-3p	0.4752
RP11-284N8.3	lincRNA	2.7922973	hsa-miR-27a-3p	0.8939

RP11-284N8.3	lincRNA	2.7922973	hsa-miR-23b-3p	0.8808
RP11-284N8.3	lincRNA	2.7922973	hsa-miR-101-3p	0.7606
RP11-284N8.3	lincRNA	2.7922973	hsa-miR-23a-3p	0.4752
RP11-284N8.3	lincRNA	2.7922973	hsa-miR-26a-5p	0.7311
RP11-284N8.3	lincRNA	2.7922973	hsa-miR-27b-3p	0.8939
RP11-284N8.3	lincRNA	2.7922973	hsa-miR-485-3p	0.4752
RP11-284N8.3	lincRNA	2.7922973	hsa-miR-338-3p	0.8589
RP11-284N8.3	lincRNA	2.7922973	hsa-miR-9-3p	0.4752
RP4-647J21.1	sense_overlapping	2.7136046	hsa-miR-21-3p	0.4752
RP11-705C15.5	processed_transcript	2.6732176	hsa-miR-196a-5p	0.4752
SSTR5-AS1	processed_transcript	2.6044732	hsa-miR-146b-5p	0.7311
LLNLR-470E3.1	sense_intronic	2.588432	hsa-miR-142-5p	0.4752
CTD-2547L24.3	processed_transcript	2.5061099	hsa-miR-708-5p	0.1097
CTD-2547L24.3	processed_transcript	2.5061099	hsa-miR-590-5p	0.4752
CTD-2540L5.6	lincRNA	2.3214336	hsa-let-7g-3p	0.4752
CTD-2540L5.6	lincRNA	2.3214336	hsa-miR-214-5p	NA
CTD-2540L5.6	lincRNA	2.3214336	hsa-miR-145-5p	0.4752
RP11-73M14.1	lincRNA	2.1991991	hsa-miR-27a-3p	0.8939
RP11-73M14.1	lincRNA	2.1991991	hsa-miR-27b-3p	0.8939
LINC00299	lincRNA	2.162583	hsa-miR-26a-5p	0.7311
AC011747.4	lincRNA	2.0105333	hsa-miR-149-5p	0.8808
AC011747.4	lincRNA	2.0105333	hsa-miR-192-5p	0.4752
HCP5	sense_overlapping	1.8474623	hsa-miR-92a-3p	0.7606
HCP5	sense_overlapping	1.8474623	hsa-miR-205-5p	0.7606
HCP5	sense_overlapping	1.8474623	hsa-miR-141-3p	0.8939
HCP5	sense_overlapping	1.8474623	hsa-miR-145-5p	0.4752
HCP5	sense_overlapping	1.8474623	hsa-miR-101-3p	0.7606
HCP5	sense_overlapping	1.8474623	hsa-miR-125a-3p	0.1097
BHLHE40-AS1	antisense	1.7920622	hsa-miR-34a-5p	0.8939

RP11-79H23.3	lincRNA	1.7292061	hsa-miR-361-5p	0.4752
RP11-79H23.3	lincRNA	1.7292061	hsa-miR-181a-5p	0.8939
RP11-79H23.3	lincRNA	1.7292061	hsa-miR-140-3p	0.4752
RP11-79H23.3	lincRNA	1.7292061	hsa-miR-27a-3p	0.8939
RP11-79H23.3	lincRNA	1.7292061	hsa-miR-23b-3p	0.8808
RP11-79H23.3	lincRNA	1.7292061	hsa-miR-181b-5p	0.4752
RP11-79H23.3	lincRNA	1.7292061	hsa-miR-181c-5p	0.4752
RP11-322D14.2	antisense	1.6860918	hsa-miR-27a-3p	0.8939
RP11-322D14.2	antisense	1.6860918	hsa-miR-27b-3p	0.8939
RP11-728G15.1	antisense	1.5885336	hsa-miR-24-3p	0.4752
AC005082.12	lincRNA	1.5723361	hsa-miR-185-5p	0.8808
AC005082.12	lincRNA	1.5723361	hsa-miR-338-5p	0.1097
AC005082.12	lincRNA	1.5723361	hsa-miR-151a-3p	0.4752
AP001055.6	lincRNA	1.5576902	hsa-let-7a-5p	0.8939
AP001055.6	lincRNA	1.5576902	hsa-let-7e-5p	0.4752
AP001055.6	lincRNA	1.5576902	hsa-let-7i-5p	0.4752
AP001055.6	lincRNA	1.5576902	hsa-let-7b-5p	0.4752
RP1-111D6.3	lincRNA	1.394617	hsa-miR-126-5p	0.4752
RP1-111D6.3	lincRNA	1.394617	hsa-miR-218-5p	0.7311
RP1-111D6.3	lincRNA	1.394617	hsa-miR-9-5p	0.8808
PRKCQ-AS1	processed_transcript	1.3781775	hsa-miR-19a-3p	0.8808
PRKCQ-AS1	processed_transcript	1.3781775	hsa-miR-19b-3p	0.4752
PRKCQ-AS1	processed_transcript	1.3781775	hsa-miR-9-5p	0.8808
LINC01197	lincRNA	1.2978823	hsa-miR-1-3p	0.4752
LINC01197	lincRNA	1.2978823	hsa-miR-137	0.8939
LINC01197	lincRNA	1.2978823	hsa-miR-28-5p	0.8808
RP3-431P23.5	lincRNA	1.2978078	hsa-miR-27a-3p	0.8939
RP3-431P23.5	lincRNA	1.2978078	hsa-miR-27b-3p	0.8939
RP1-60O19.1	lincRNA	1.2316637	hsa-miR-195-5p	0.4752

RP1-60O19.1	lincRNA	1.2316637	hsa-miR-199a-5p	0.8808
RP1-60O19.1	lincRNA	1.2316637	hsa-miR-150-5p	0.4752
RP1-60O19.1	lincRNA	1.2316637	hsa-miR-218-5p	0.7311
RP1-60O19.1	lincRNA	1.2316637	hsa-miR-15a-5p	0.8939
RP1-60O19.1	lincRNA	1.2316637	hsa-miR-15b-5p	0.8808
RP1-60O19.1	lincRNA	1.2316637	hsa-miR-16-5p	0.7606
RP1-60O19.1	lincRNA	1.2316637	hsa-miR-29b-1-5p	0.4752
RP1-60O19.1	lincRNA	1.2316637	hsa-miR-145-5p	0.4752
RP1-60O19.1	lincRNA	1.2316637	hsa-miR-214-3p	0.7606
RP1-60O19.1	lincRNA	1.2316637	hsa-miR-338-3p	0.8589
RP11-203J24.9	processed_transcript	1.226269	hsa-let-7g-3p	0.4752
RP11-203J24.9	processed_transcript	1.226269	hsa-miR-19a-3p	0.8808
RP11-203J24.9	processed_transcript	1.226269	hsa-miR-19b-3p	0.4752
RP11-203J24.9	processed_transcript	1.226269	hsa-miR-188-5p	0.4752
CTD-2369P2.8	antisense	1.1793339	hsa-miR-92a-3p	0.7606
CTD-2369P2.8	antisense	1.1793339	hsa-miR-25-3p	0.8589
CTD-2369P2.8	antisense	1.1793339	hsa-miR-32-5p	0.4752
CTD-2369P2.8	antisense	1.1793339	hsa-miR-363-3p	0.4752
CTD-2369P2.8	antisense	1.1793339	hsa-miR-194-5p	0.4752
CTD-2369P2.8	antisense	1.1793339	hsa-miR-107	0.8808
EPHA1-AS1	antisense	1.1655127	hsa-miR-532-5p	0.8808
RP11-147L13.8	lincRNA	1.0226666	hsa-miR-221-3p	0.8808
RP11-147L13.8	lincRNA	1.0226666	hsa-miR-185-5p	0.8808
FZD10-AS1	processed_transcript	1.0135296	hsa-miR-9-5p	0.8808
RP11-588K22.2	sense_overlapping	1.0105129	hsa-miR-361-5p	0.4752
RP11-588K22.2	sense_overlapping	1.0105129	hsa-miR-195-5p	0.4752
RP11-588K22.2	sense_overlapping	1.0105129	hsa-let-7e-5p	0.4752
RP11-588K22.2	sense_overlapping	1.0105129	hsa-miR-21-5p	0.7606
RP11-588K22.2	sense_overlapping	1.0105129	hsa-let-7i-5p	0.4752

RP11-588K22.2	sense_overlapping	1.0105129	hsa-miR-19a-3p	0.8808
RP11-588K22.2	sense_overlapping	1.0105129	hsa-miR-19b-3p	0.4752
RP11-588K22.2	sense_overlapping	1.0105129	hsa-miR-9-5p	0.8808
RP11-588K22.2	sense_overlapping	1.0105129	hsa-miR-34a-5p	0.8939
RP11-588K22.2	sense_overlapping	1.0105129	hsa-let-7a-5p	0.8939
RP11-588K22.2	sense_overlapping	1.0105129	hsa-let-7b-5p	0.4752
RP11-588K22.2	sense_overlapping	1.0105129	hsa-let-7f-5p	0.4752
RP11-588K22.2	sense_overlapping	1.0105129	hsa-miR-15a-5p	0.8939
RP11-588K22.2	sense_overlapping	1.0105129	hsa-miR-126-5p	0.4752
RP11-588K22.2	sense_overlapping	1.0105129	hsa-miR-454-3p	0.4752
RP11-588K22.2	sense_overlapping	1.0105129	hsa-miR-16-5p	0.7606
RP11-588K22.2	sense_overlapping	1.0105129	hsa-miR-182-5p	0.8808
RP11-588K22.2	sense_overlapping	1.0105129	hsa-miR-98-5p	0.7606
RP11-588K22.2	sense_overlapping	1.0105129	hsa-miR-135a-5p	0.8965
RP11-588K22.2	sense_overlapping	1.0105129	hsa-let-7c-5p	0.4752
RP11-588K22.2	sense_overlapping	1.0105129	hsa-miR-301b-3p	0.4752
CTD-2377D24.6	lincRNA	1.0104761	hsa-miR-361-5p	0.4752
CTD-2377D24.6	lincRNA	1.0104761	hsa-miR-26a-5p	0.7311
RP11-326I11.5	sense_intronic	1.0075254	hsa-miR-338-3p	0.8589
RP11-54O7.1	lincRNA	-1.104466	hsa-miR-16-5p	0.7606
RP11-54O7.1	lincRNA	-1.104466	hsa-miR-24-3p	0.4752
RP11-54O7.1	lincRNA	-1.104466	hsa-miR-9-5p	0.8808
RP11-54O7.1	lincRNA	-1.104466	hsa-miR-107	0.8808
RP11-54O7.1	lincRNA	-1.104466	hsa-miR-424-5p	0.4752
RP11-707A18.1	lincRNA	-1.145131	hsa-miR-181a-5p	0.8939
RP11-115D19.1	antisense	-1.19958	hsa-miR-9-5p	0.8808
RP11-74E22.6	lincRNA	-1.24046	hsa-miR-125a-3p	0.1097
RP6-24A23.7	sense_overlapping	-1.27855	hsa-miR-24-3p	0.4752
RP6-24A23.7	sense_overlapping	-1.27855	hsa-miR-34a-5p	0.8939

RP6-24A23.7	sense_overlapping	-1.27855	hsa-miR-34b-5p	0.8808
RP6-24A23.7	sense_overlapping	-1.27855	hsa-miR-34c-5p	0.8939
RP6-24A23.7	sense_overlapping	-1.27855	hsa-miR-449a	0.4752
RP6-24A23.7	sense_overlapping	-1.27855	hsa-miR-629-5p	0.4752
RP6-24A23.7	sense_overlapping	-1.27855	hsa-miR-15a-5p	0.8939
RP6-24A23.7	sense_overlapping	-1.27855	hsa-miR-16-5p	0.7606
RP6-24A23.7	sense_overlapping	-1.27855	hsa-miR-491-5p	0.4752
RP6-24A23.7	sense_overlapping	-1.27855	hsa-miR-144-3p	0.8808
RP6-24A23.7	sense_overlapping	-1.27855	hsa-miR-29a-3p	0.8589
RP6-24A23.7	sense_overlapping	-1.27855	hsa-miR-29b-3p	0.8939
RP6-24A23.7	sense_overlapping	-1.27855	hsa-miR-29c-3p	0.4752
RP11-13K12.1	lincRNA	-1.329828	hsa-miR-338-3p	0.8589
AC138430.4	processed_transcript	-1.39162	hsa-miR-100-5p	0.8939
AC138430.4	processed_transcript	-1.39162	hsa-miR-99a-5p	0.4752
RP3-523K23.2	sense_overlapping	-1.400005	hsa-miR-19a-3p	0.8808
RP3-523K23.2	sense_overlapping	-1.400005	hsa-miR-182-5p	0.8808
RP6-191P20.4	antisense	-1.496962	hsa-miR-181a-5p	0.8939
LINC01391	lincRNA	-1.517676	hsa-miR-34c-5p	0.8939
DPP10-AS1	antisense	-1.529026	hsa-miR-9-5p	0.8808
UG0898H09	lincRNA	-1.649409	hsa-miR-221-3p	0.8808
UG0898H09	lincRNA	-1.649409	hsa-miR-137	0.8939
UG0898H09	lincRNA	-1.649409	hsa-miR-218-5p	0.7311
CTC-471C19.1	lincRNA	-2.259944	hsa-miR-27a-3p	0.8939
CTC-471C19.1	lincRNA	-2.259944	hsa-miR-27b-3p	0.8939
FASLG	protein_coding	3.3157376	hsa-miR-21-3p	0.4752
CD274	protein_coding	2.1936648	hsa-miR-708-5p	0.1097
BTG2	protein_coding	1.1490555	hsa-miR-590-5p	0.4752
SOCS1	protein_coding	2.1563027	hsa-miR-142-5p	0.4752
CYBB	protein_coding	2.530698	hsa-miR-148b-3p	0.9578

CSF1	protein_coding	1.3871534	hsa-miR-148b-3p	0.9578
WNT1	protein_coding	1.7087148	hsa-miR-148b-3p	0.9578
CORO1A	protein_coding	3.0892221	hsa-miR-24-3p	0.4752
FASLG	protein_coding	3.3157376	hsa-miR-149-5p	0.8808
IFNG	protein_coding	3.7412393	hsa-miR-24-3p	0.4752
PTGER2	protein_coding	2.0621902	hsa-miR-149-5p	0.8808
MAFB	protein_coding	1.0798385	hsa-miR-24-3p	0.4752
REG4	protein_coding	3.3794795	hsa-miR-24-3p	0.4752
CYP11B2	protein_coding	3.7037909	hsa-miR-24-3p	0.4752
GATA3	protein_coding	1.0454894	hsa-miR-24-3p	0.4752
CD28	protein_coding	2.0955913	hsa-miR-145-5p	0.4752
FLI1	protein_coding	1.64209	hsa-miR-145-5p	0.4752
CD40	protein_coding	1.4220723	hsa-miR-145-5p	0.4752
NAIP	protein_coding	1.2888904	hsa-miR-145-5p	0.4752
CAMK1D	protein_coding	1.3173515	hsa-miR-145-5p	0.4752
MMP12	protein_coding	2.0239601	hsa-miR-145-5p	0.4752
MIXL1	protein_coding	1.3814066	hsa-miR-145-5p	0.4752
SERPINE1	protein_coding	1.3677744	hsa-miR-145-5p	0.4752
FGF9	protein_coding	-1.565606	hsa-miR-140-5p	0.8939
NLRC5	protein_coding	1.6376025	hsa-miR-34a-5p	0.8939
TREM2	protein_coding	1.7815268	hsa-miR-34a-5p	0.8939
CXCR6	protein_coding	3.0669866	hsa-miR-361-5p	0.4752
PTPN7	protein_coding	2.6421109	hsa-miR-126-5p	0.4752
ALOX5	protein_coding	2.1516476	hsa-miR-19a-3p	0.8808
SOCS1	protein_coding	2.1563027	hsa-miR-19a-3p	0.8808
SOCS1	protein_coding	2.1563027	hsa-miR-19b-3p	0.4752
SIGLEC1	protein_coding	2.0810312	hsa-miR-27a-3p	0.8939
TLR7	protein_coding	2.388593	hsa-miR-19a-3p	0.8808
PTK2B	protein_coding	1.4865526	hsa-miR-23b-3p	0.8808

IFNG	protein_coding	3.7412393	hsa-miR-27a-3p	0.8939
PTGER4	protein_coding	1.8085852	hsa-miR-101-3p	0.7606
CXCL12	protein_coding	1.7078623	hsa-miR-126-5p	0.4752
CXCL12	protein_coding	1.7078623	hsa-miR-23a-3p	0.4752
ST8SIA4	protein_coding	1.5284402	hsa-miR-26a-5p	0.7311
MRC1	protein_coding	1.6736868	hsa-miR-27a-3p	0.8939
PIK3CG	protein_coding	1.9285215	hsa-miR-26a-5p	0.7311
PIK3CG	protein_coding	1.9285215	hsa-miR-27a-3p	0.8939
VEGFC	protein_coding	1.2642738	hsa-miR-101-3p	0.7606
VEGFC	protein_coding	1.2642738	hsa-miR-27b-3p	0.8939
SLC40A1	protein_coding	1.2699472	hsa-miR-485-3p	0.4752
MMP9	protein_coding	1.6731235	hsa-miR-338-3p	0.8589
MMP9	protein_coding	1.6731235	hsa-miR-9-3p	0.4752
GATA3	protein_coding	1.0454894	hsa-miR-27a-3p	0.8939
CD22	protein_coding	1.0141539	hsa-miR-19a-3p	0.8808
PRKAA2	protein_coding	-1.141555	hsa-miR-27a-3p	0.8939
FGF9	protein_coding	-1.565606	hsa-miR-26a-5p	0.7311
INPP5D	protein_coding	2.5499579	hsa-miR-155-5p	0.7606
SPI1	protein_coding	2.2611973	hsa-miR-155-5p	0.7606
SPI1	protein_coding	2.2611973	hsa-miR-34a-5p	0.8939
CARD11	protein_coding	2.9837486	hsa-miR-155-5p	0.7606
CYBB	protein_coding	2.530698	hsa-miR-34a-5p	0.8939
FLI1	protein_coding	1.64209	hsa-miR-155-5p	0.7606
ARHGDIB	protein_coding	1.5952346	hsa-miR-34a-5p	0.8939
ZAP70	protein_coding	2.3805117	hsa-miR-34a-5p	0.8939
GCSAM	protein_coding	2.1224941	hsa-miR-155-5p	0.7606
NR1H3	protein_coding	1.1328536	hsa-miR-155-5p	0.7606
STAT1	protein_coding	1.359901	hsa-miR-155-5p	0.7606
SAMHD1	protein_coding	1.3160648	hsa-miR-155-5p	0.7606

PIK3CG	protein_coding	1.9285215	hsa-miR-34a-5p	0.8939
TM6SF1	protein_coding	1.2968658	hsa-miR-155-5p	0.7606
CCL22	protein_coding	1.9343022	hsa-miR-34a-5p	0.8939
MAFB	protein_coding	1.0798385	hsa-miR-155-5p	0.7606
OLR1	protein_coding	1.8100657	hsa-miR-155-5p	0.7606
CCND2	protein_coding	1.4486746	hsa-miR-124-3p	0.8808
CCND2	protein_coding	1.4486746	hsa-miR-155-5p	0.7606
CD68	protein_coding	1.0045876	hsa-miR-155-5p	0.7606
FGF7	protein_coding	1.1384955	hsa-miR-155-5p	0.7606
GDF5	protein_coding	1.311298	hsa-miR-34a-5p	0.8939
SELE	protein_coding	1.1095313	hsa-miR-155-5p	0.7606
KCNH2	protein_coding	-1.009973	hsa-miR-34a-5p	0.8939
IKZF1	protein_coding	3.1848992	hsa-miR-92a-3p	0.7606
IL32	protein_coding	2.7802845	hsa-miR-205-5p	0.7606
STAT4	protein_coding	2.1733972	hsa-miR-141-3p	0.8939
CD69	protein_coding	3.0289513	hsa-miR-92a-3p	0.7606
PRDM1	protein_coding	1.8497658	hsa-miR-101-3p	0.7606
PRDM1	protein_coding	1.8497658	hsa-miR-125a-3p	0.1097
CCL8	protein_coding	2.4065419	hsa-miR-92a-3p	0.7606
IL23R	protein_coding	1.084645	hsa-miR-125a-3p	0.1097
LSP1	protein_coding	2.3266976	hsa-miR-196a-5p	0.4752
CCR7	protein_coding	3.5802069	hsa-let-7a-5p	0.8939
FASLG	protein_coding	3.3157376	hsa-let-7e-5p	0.4752
SOCS1	protein_coding	2.1563027	hsa-let-7i-5p	0.4752
IL2	protein_coding	2.4669726	hsa-let-7i-5p	0.4752
CCND2	protein_coding	1.4486746	hsa-let-7a-5p	0.8939
CCND2	protein_coding	1.4486746	hsa-let-7b-5p	0.4752
WNT1	protein_coding	1.7087148	hsa-let-7a-5p	0.8939
WNT1	protein_coding	1.7087148	hsa-let-7e-5p	0.4752

TLR4	protein_coding	1.028326	hsa-let-7b-5p	0.4752
TLR4	protein_coding	1.028326	hsa-let-7i-5p	0.4752
IL10RA	protein_coding	2.5638693	hsa-miR-185-5p	0.8808
PLAC8	protein_coding	3.0771871	hsa-miR-185-5p	0.8808
SLC8A1	protein_coding	1.7588832	hsa-miR-185-5p	0.8808
TG	protein_coding	-3.110697	hsa-miR-185-5p	0.8808
EPHB2	protein_coding	1.152284	hsa-miR-185-5p	0.8808
EFEMP1	protein_coding	1.1137182	hsa-miR-338-5p	0.1097
NTRK3	protein_coding	-1.170012	hsa-miR-151a-3p	0.4752
NTRK3	protein_coding	-1.170012	hsa-miR-185-5p	0.8808
CCL5	protein_coding	3.3597451	hsa-let-7g-3p	0.4752
IL10	protein_coding	1.111578	hsa-miR-19a-3p	0.8808
GREM2	protein_coding	-1.005495	hsa-miR-188-5p	0.4752
PTPN22	protein_coding	2.775133	hsa-miR-181a-5p	0.8939
CD4	protein_coding	2.1443353	hsa-miR-181a-5p	0.8939
CD38	protein_coding	2.999445	hsa-miR-140-3p	0.4752
IFNG	protein_coding	3.7412393	hsa-miR-181a-5p	0.8939
TCL1A	protein_coding	4.6611426	hsa-miR-181b-5p	0.4752
ST8SIA4	protein_coding	1.5284402	hsa-miR-181c-5p	0.4752
SAMHD1	protein_coding	1.3160648	hsa-miR-181a-5p	0.8939
IL2	protein_coding	2.4669726	hsa-miR-181c-5p	0.4752
RASSF6	protein_coding	1.910464	hsa-miR-181a-5p	0.8939
RGS16	protein_coding	1.030616	hsa-miR-181a-5p	0.8939
TMSB4X	protein_coding	1.7833113	hsa-miR-1-3p	0.4752
SLC8A1	protein_coding	1.7588832	hsa-miR-1-3p	0.4752
CXCL12	protein_coding	1.7078623	hsa-miR-137	0.8939
CXCL12	protein_coding	1.7078623	hsa-miR-1-3p	0.4752
IL34	protein_coding	2.0150641	hsa-miR-28-5p	0.8808
CEBPA	protein_coding	1.379097	hsa-miR-1-3p	0.4752

SERPINE1	protein_coding	1.3677744	hsa-miR-192-5p	0.4752
SCN5A	protein_coding	1.4188237	hsa-miR-192-5p	0.4752
TNFSF10	protein_coding	1.735215	hsa-miR-25-3p	0.8589
BTG2	protein_coding	1.1490555	hsa-miR-32-5p	0.4752
REG4	protein_coding	3.3794795	hsa-miR-363-3p	0.4752
CCL26	protein_coding	1.2329738	hsa-miR-25-3p	0.8589
IL10	protein_coding	1.111578	hsa-miR-194-5p	0.4752
CHGA	protein_coding	-1.294564	hsa-miR-107	0.8808
DSC2	protein_coding	-1.193547	hsa-miR-25-3p	0.8589
TP63	protein_coding	-1.182197	hsa-miR-92a-3p	0.7606
CCL4	protein_coding	2.9207343	hsa-miR-195-5p	0.4752
FASLG	protein_coding	3.3157376	hsa-miR-21-5p	0.7606
SOCS1	protein_coding	2.1563027	hsa-miR-21-5p	0.7606
TNFAIP8	protein_coding	1.8523279	hsa-miR-9-5p	0.8808
PRDM1	protein_coding	1.8497658	hsa-let-7a-5p	0.8939
PRDM1	protein_coding	1.8497658	hsa-let-7b-5p	0.4752
PRDM1	protein_coding	1.8497658	hsa-let-7f-5p	0.4752
PRDM1	protein_coding	1.8497658	hsa-miR-9-5p	0.8808
CXCL10	protein_coding	2.7879188	hsa-miR-15a-5p	0.8939
CXCL12	protein_coding	1.7078623	hsa-miR-454-3p	0.4752
CCR1	protein_coding	1.5319707	hsa-miR-21-5p	0.7606
IL12B	protein_coding	2.1596205	hsa-miR-16-5p	0.7606
ADORA2A	protein_coding	1.3126857	hsa-miR-16-5p	0.7606
CCND2	protein_coding	1.4486746	hsa-miR-15a-5p	0.8939
CCND2	protein_coding	1.4486746	hsa-miR-16-5p	0.7606
CCND2	protein_coding	1.4486746	hsa-miR-182-5p	0.8808
CCND2	protein_coding	1.4486746	hsa-miR-98-5p	0.7606
WNT1	protein_coding	1.7087148	hsa-miR-34a-5p	0.8939
SLC6A4	protein_coding	-1.287985	hsa-miR-135a-5p	0.8965

SLC6A4	protein_coding	-1.287985	hsa-miR-16-5p	0.7606
IL10	protein_coding	1.111578	hsa-let-7c-5p	0.4752
IL10	protein_coding	1.111578	hsa-miR-98-5p	0.7606
ICOSLG	protein_coding	1.2346422	hsa-miR-21-5p	0.7606
GDF5	protein_coding	1.311298	hsa-miR-21-5p	0.7606
CYP19A1	protein_coding	-1.280117	hsa-let-7f-5p	0.4752
CYP19A1	protein_coding	-1.280117	hsa-miR-19b-3p	0.4752
CYP19A1	protein_coding	-1.280117	hsa-miR-98-5p	0.7606
TCEAL7	protein_coding	1.0061387	hsa-miR-182-5p	0.8808
TP63	protein_coding	-1.182197	hsa-miR-21-5p	0.7606
TP63	protein_coding	-1.182197	hsa-miR-301b-3p	0.4752
CYP4Z1	protein_coding	-1.209784	hsa-miR-9-5p	0.8808
RASSF5	protein_coding	2.0611323	hsa-miR-214-5p	NA
HOXA9	protein_coding	-1.040714	hsa-miR-145-5p	0.4752
POU2F2	protein_coding	2.3862716	hsa-miR-9-5p	0.8808
TNF	protein_coding	2.2216054	hsa-miR-19a-3p	0.8808
BTG2	protein_coding	1.1490555	hsa-miR-27a-3p	0.8939
CYP1B1	protein_coding	1.5009887	hsa-miR-27b-3p	0.8939
CRISP2	protein_coding	-1.447602	hsa-miR-27b-3p	0.8939
SYK	protein_coding	1.0593451	hsa-miR-532-5p	0.8808
SLC5A5	protein_coding	1.9657187	hsa-miR-146b-5p	0.7311
ONECUT2	protein_coding	-1.078639	hsa-miR-9-5p	0.8808
CORO1A	protein_coding	3.0892221	hsa-miR-221-3p	0.8808
SOCS1	protein_coding	2.1563027	hsa-miR-221-3p	0.8808
CXCL12	protein_coding	1.7078623	hsa-miR-221-3p	0.8808
TNFSF10	protein_coding	1.735215	hsa-miR-221-3p	0.8808
NAIP	protein_coding	1.2888904	hsa-miR-221-3p	0.8808
CXCR4	protein_coding	1.7729261	hsa-miR-9-5p	0.8808
NTRK3	protein_coding	-1.170012	hsa-miR-9-5p	0.8808

CYP3A4	protein_coding	-1.645801	hsa-miR-27b-3p	0.8939
PAX7	protein_coding	-1.344672	hsa-miR-27b-3p	0.8939
DIRAS3	protein_coding	-1.179906	hsa-miR-221-3p	0.8808
CALN1	protein_coding	-1.704655	hsa-miR-137	0.8939
SOST	protein_coding	-1.621914	hsa-miR-218-5p	0.7311
VOPP1	protein_coding	1.0493781	hsa-miR-218-5p	0.7311
DRD2	protein_coding	-1.77275	hsa-miR-9-5p	0.8808
GRM7	protein_coding	-1.330442	hsa-miR-34c-5p	0.8939
HNF4A	protein_coding	-1.083016	hsa-miR-34c-5p	0.8939
FGFR3	protein_coding	-2.267366	hsa-miR-100-5p	0.8939
FGFR3	protein_coding	-2.267366	hsa-miR-99a-5p	0.4752
WIF1	protein_coding	-1.476228	hsa-miR-181a-5p	0.8939
SLC6A4	protein_coding	-1.287985	hsa-miR-24-3p	0.4752
CDX2	protein_coding	-1.55935	hsa-miR-424-5p	0.4752
CDX2	protein_coding	-1.55935	hsa-miR-9-5p	0.8808
HNF4A	protein_coding	-1.083016	hsa-miR-24-3p	0.4752
HNF4A	protein_coding	-1.083016	hsa-miR-34a-5p	0.8939
HNF4A	protein_coding	-1.083016	hsa-miR-34b-5p	0.8808
HNF4A	protein_coding	-1.083016	hsa-miR-449a	0.4752
HNF4A	protein_coding	-1.083016	hsa-miR-629-5p	0.4752
WNT3A	protein_coding	-1.018105	hsa-miR-15a-5p	0.8939
WNT3A	protein_coding	-1.018105	hsa-miR-16-5p	0.7606
WNT3A	protein_coding	-1.018105	hsa-miR-491-5p	0.4752
FGA	protein_coding	-1.831016	hsa-miR-144-3p	0.8808
FGA	protein_coding	-1.831016	hsa-miR-29a-3p	0.8589
FGA	protein_coding	-1.831016	hsa-miR-29b-3p	0.8939
FGA	protein_coding	-1.831016	hsa-miR-29c-3p	0.4752
CCR7	protein_coding	3.5802069	hsa-miR-199a-5p	0.8808
FLT3	protein_coding	2.8230616	hsa-miR-150-5p	0.4752

POU2F2	protein_coding	2.3862716	hsa-miR-218-5p	0.7311
IFNG	protein_coding	3.7412393	hsa-miR-15a-5p	0.8939
IFNG	protein_coding	3.7412393	hsa-miR-15b-5p	0.8808
IFNG	protein_coding	3.7412393	hsa-miR-16-5p	0.7606
JAK3	protein_coding	1.8745406	hsa-miR-29b-1-5p	0.4752
SEMA4D	protein_coding	1.4811331	hsa-miR-214-3p	0.7606
ALPK2	protein_coding	1.7165143	hsa-miR-214-3p	0.7606
COL4A4	protein_coding	1.451796	hsa-miR-150-5p	0.4752
LTF	protein_coding	2.2861861	hsa-miR-214-3p	0.7606
MMP9	protein_coding	1.6731235	hsa-miR-15b-5p	0.8808
DDC	protein_coding	-1.093134	hsa-miR-145-5p	0.4752
FOXF2	protein_coding	-1.029889	hsa-miR-182-5p	0.8808
CDX2	protein_coding	-1.55935	hsa-miR-181a-5p	0.8939

ceRNA, competing endogenous RNA; CM, cutaneous melanoma. †: biotypes of the ceRNAs denoted by GENCODE; ‡: log2 fold change of the expression of ceRNAs between tumors with higher immune scores and those with lower immune scores; §: confidence scores assigned by Mammal NcRNA-Disease Repository (MNDR) V2.0 (http://www.rna-society.org/mndr/) to describe the reliability of the association between miRNAs and melanoma or skin melanoma, NA means not available.

Table S6. The prognostic genes validated in the independent dataset.

Gene symbol	prognostic values†	target type‡	PPI network degree§	module identity¶
PTPRC	protective	Clinical Trial target	45	module3
CD86	protective	Successful target	39	module3
LCK	protective	Successful target	30	module1
LILRB2	protective	NA	27	module1
TLR8	protective	Clinical Trial target	25	module1
CD40LG	protective	Clinical Trial target	25	module2

CD3E	protective	Successful target	23	module1
CD274	protective	Successful	22	module1
MNDA	protective	NA	20	module1
CTSS	protective	Clinical Trial target	20	module1
HLA-DRA	protective	NA	19	module1
CXCR3	protective	Clinical Trial target	19	module1
FCER1G	protective	Successful target	18	module1
HLA-B	protective	Clinical Trial target	17	module1
HLA-C	protective	NA	17	module1
PDCD1	protective	NA	17	module1
HLA-DQB2	protective	NA	16	module1
HLA-DPA1	protective	NA	16	module1
CD74	protective	Clinical Trial target	16	module1
HCK	protective	Research target	16	module1
HLA-DRB5	protective	Clinical Trial target	16	module1
SLAMF1	protective	NA	15	module1
NCF2	protective	NA	15	module1
NCR3	protective	NA	15	NA
CD79A	protective	NA	15	NA
HLA-F	protective	NA	14	module1
C3AR1	protective	Research target	14	module1
CD52	protective	Successful target	14	module1
BTLA	protective	NA	14	NA
CLEC7A	protective	NA	14	module2
IGSF6	protective	NA	14	module2
PDCD1LG2	protective	NA	13	module1
CD5	protective	Research target	13	module1
FASLG	protective	Research target	13	module1
CD163	protective	Research target	13	module2

MS4A1	protective	Successful target	11	NA
SLAMF6	protective	Research target	11	NA
NOD2	protective	Clinical Trial target	10	module2
CLEC4A	protective	NA	10	module2
TLR10	protective	NA	10	module2
IL21R	protective	Clinical Trial target	10	module4
LY9	protective	Research target	10	module4
POU2AF1	protective	NA	9	NA
HLA-DOA	protective	NA	8	module1
KIR2DL3	protective	Clinical Trial target	8	NA
LTA	protective	NA	8	NA
KIR2DL4	protective	NA	8	NA
CARD11	protective	NA	7	NA
KIR3DL1	protective	NA	7	NA
P2RY10	protective	Research target	7	module4
SRGN	protective	NA	6	NA
TNFRSF17	protective	Research target	6	NA
VNN2	protective	NA	6	NA
LYZ	protective	Research target	6	module3
P2RY13	protective	Research target	5	NA
IL12RB1	protective	NA	5	NA
IGJ	protective	NA	5	NA
LAIR1	protective	NA	5	NA
FLT3LG	protective	NA	5	module3
CST7	protective	NA	4	NA
IGLL1	protective	NA	4	NA
SLC7A7	protective	NA	4	NA
MGC29506	protective	NA	4	NA
SERPINA1	protective	Successful target	4	NA

PTK2B	protective	Research target	3	NA
LAMP3	protective	NA	3	NA
ARHGAP25	protective	NA	3	NA
AMICA1	protective	NA	3	NA
CASP5	protective	Research target	3	NA
LAX1	protective	NA	2	NA
ZBP1	protective	NA	2	NA
BCL11B	protective	NA	2	NA
LST1	protective	NA	2	NA
RASGRP1	protective	NA	2	NA
GZMK	protective	NA	2	NA
GCH1	protective	Clinical Trial target	1	NA
UBD	protective	NA	1	NA
HSH2D	protective	NA	1	NA
C6orf190	protective	NA	1	NA
C2	protective	NA	1	NA
PIM2	protective	Clinical Trial target	1	NA
FLVCR2	protective	NA	1	NA
TIMD4	protective	NA	1	NA
ACSL5	protective	NA	1	NA
BTN3A3	protective	NA	NA	NA
ASCL2	protective	NA	NA	NA
PSD4	protective	NA	NA	NA
JSRP1	protective	NA	NA	NA
FAM105A	protective	NA	NA	NA
APOBEC3G	protective	NA	NA	NA
SIRPG	protective	NA	NA	NA
IL4I1	protective	NA	NA	NA
ILDR1	protective	NA	NA	NA

TNFAIP8	protective	NA	NA	NA
LINCR	protective	NA	NA	NA
ZNF831	protective	NA	NA	NA
ZMYND15	protective	NA	NA	NA
SUSD3	protective	NA	NA	NA
MEI1	protective	NA	NA	NA
SAMD3	protective	NA	NA	NA
TBC1D10C	protective	NA	NA	NA
SEMA4A	protective	NA	NA	NA
ZBED2	protective	NA	NA	NA
FCRL3	protective	NA	NA	NA
SQRDL	protective	NA	NA	NA
FAM159A	protective	NA	NA	NA
ACAP1	protective	NA	NA	NA
FAM113B	protective	NA	NA	NA
FLJ16686	protective	NA	NA	NA

†: protective means the expression of the gene is positively correlated with patient survival time; ‡: type of the drug target from Therapeutic target database; §: degree of the nodes in protein-protein interaction network, NA means not available in the network; ¶: module that the node belongs to, NA means not available in any module.

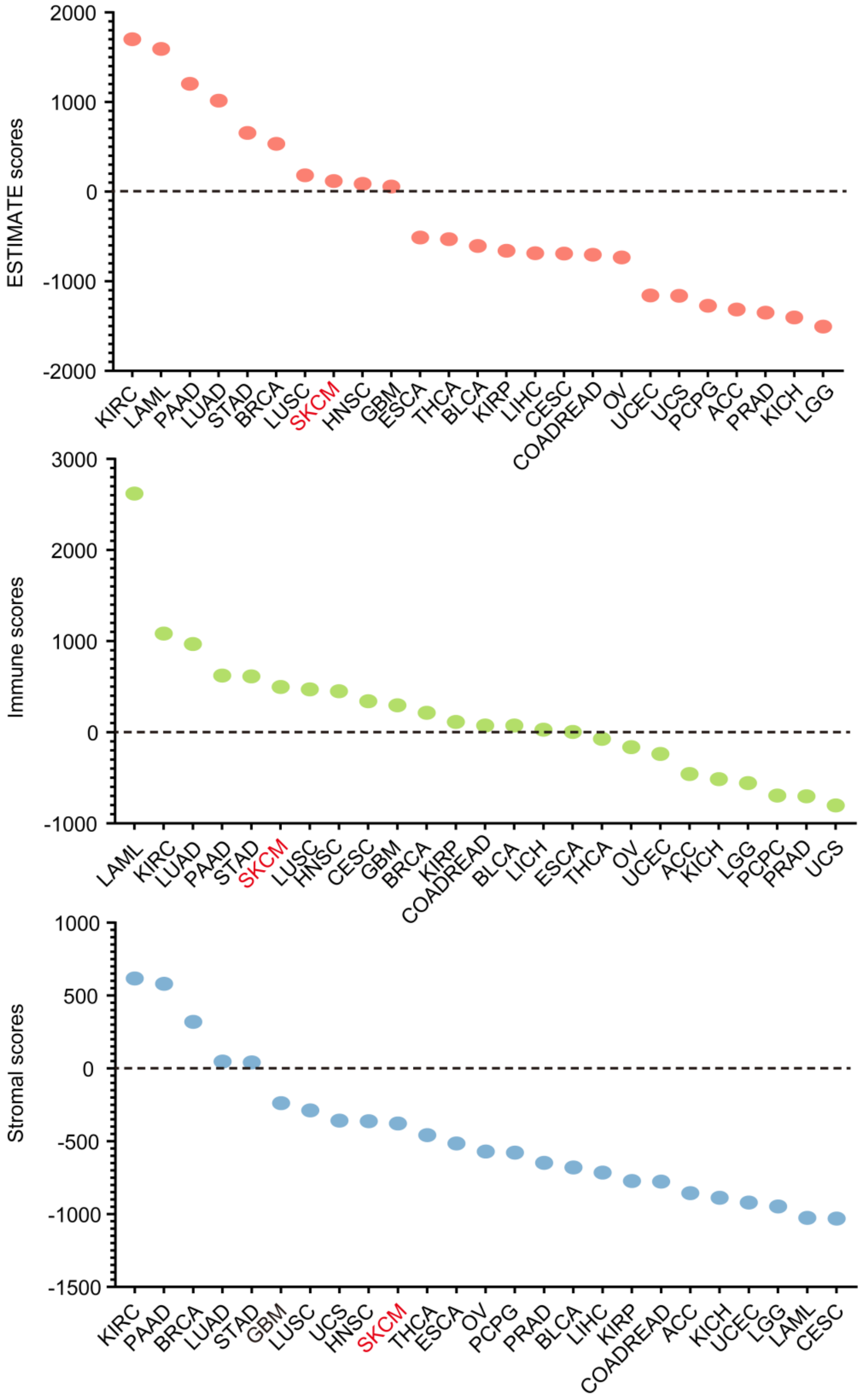

Figure S1. Average ESTIMATE, immune and stromal scores across 25 TCGA cancer types. TCGA, The Cancer Genome Atlas; LAML, Acute Myeloid Leukemia; ACC, Adrenocortical carcinoma; BLCA, Bladder Urothelial Carcinoma; LGG, Brain Lower

Grade Glioma; BRCA, Breast invasive carcinoma; CESC, Cervical squamous cell carcinoma and endocervical adenocarcinoma; COADREAD, Colon adenocarcinoma and Rectum adenocarcinoma; ESCA, Esophageal carcinoma; GBM, Glioblastoma multiforme; HNSC, Head and Neck squamous cell carcinoma; KICH, Kidney Chromophobe; KIRC, Kidney renal clear cell carcinoma; KIRP, Kidney renal papillary cell carcinoma; LIHC, Liver hepatocellular carcinoma; LUAD, Lung adenocarcinoma; LUSC, Lung squamous cell carcinoma; OV, Ovarian serous cystadenocarcinoma; PAAD, Pancreatic adenocarcinoma; PCPG, Pheochromocytoma and Paraganglioma; PRAD, Prostate adenocarcinoma; SKCM, skin cutaneous melanoma; STAD, Stomach adenocarcinoma; THCA, Thyroid carcinoma; UCS, Uterine Carcinosarcoma; UCEC, Uterine Corpus Endometrial Carcinoma.

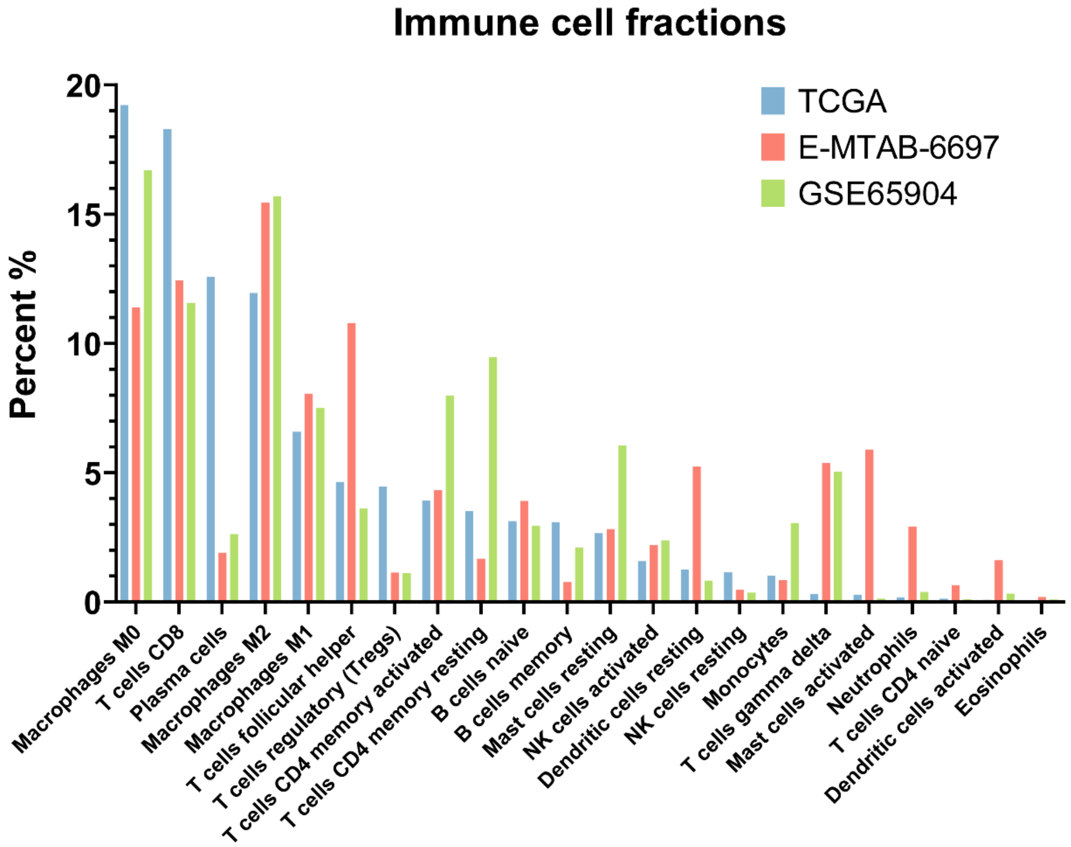

Figure S2. The fractions of 22 types of immune cell subsets in CM based on TCGA, ArrayExpress and GEO datasets. CM, cutaneous melanoma.

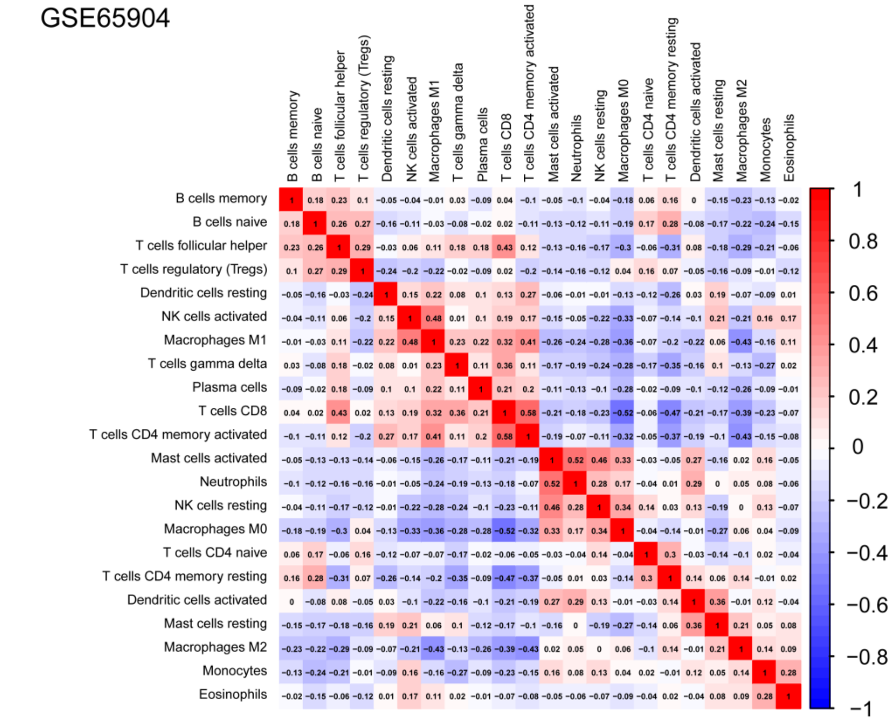

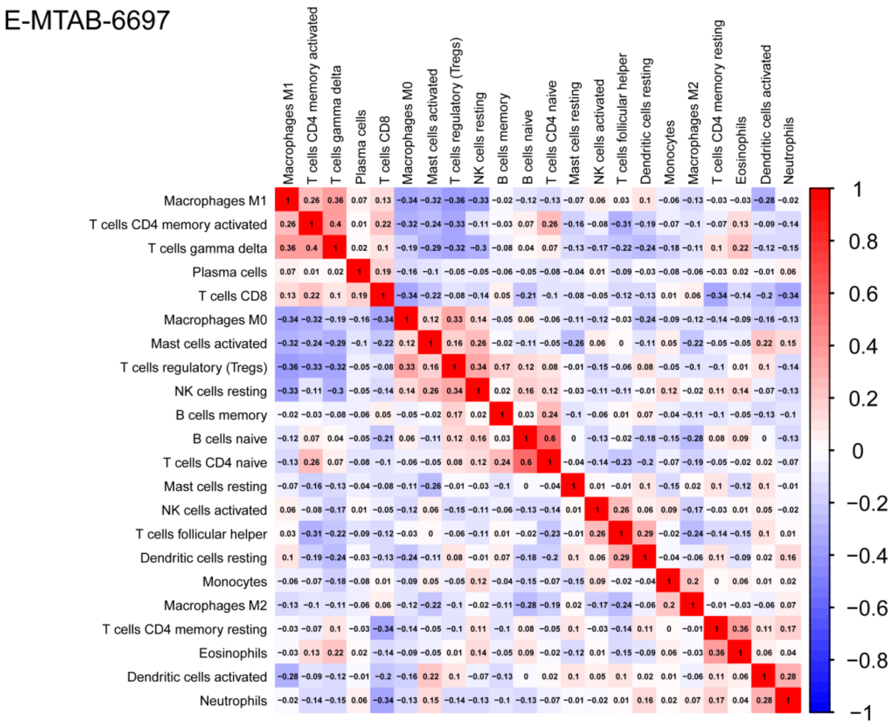

Figure S3. Correlation of the 22 types of immune cell subsets in CM based on GEO and ArrayExpress datasets. CM, cutaneous melanoma.

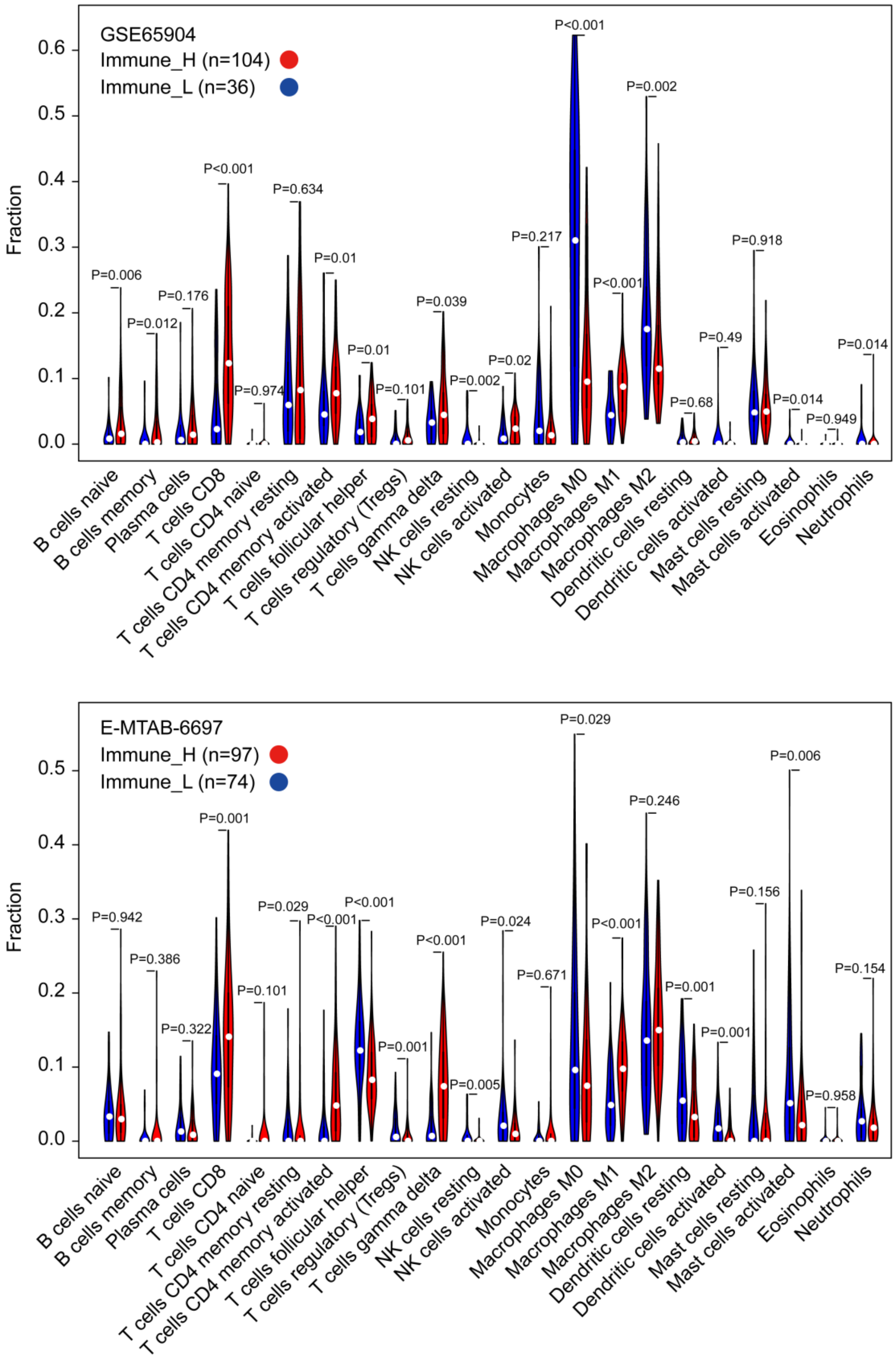

Figure S4. Comparison of the immune cell fractions between tumor tissues of the immune_H and immune_L groups based on GEO and ArrayExpress datasets. For the GSE65904 dataset, 104 samples from the immune_H group and 36 samples from the immune_L group were successfully deconvolved by CIBERSORT (P<0.05). For the E-MTAB-6697 dataset, 97 samples from the immune_H group and 74 samples from the

immune_L group were successfully deconvolved (P<0.05).

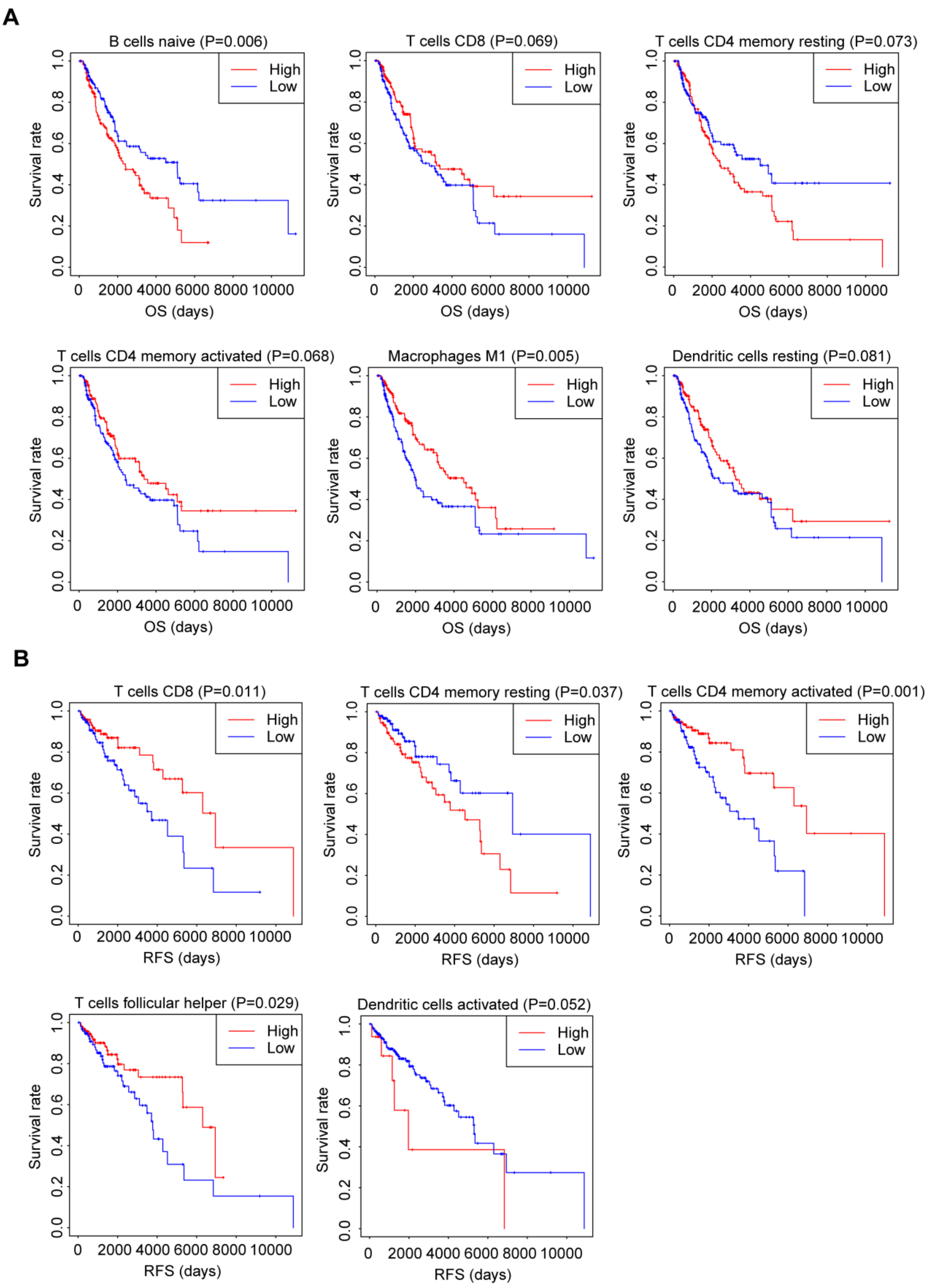

Figure S5. Kaplan-Meier analysis of the immune cell subsets for (A) OS and (B) RFS in CM. The survival rates were estimated based on the TCGA data, and only the cells

with a log-rank P-value<0.1 were displayed. OS, overall survival; RFS, relapse-free survival; CM, cutaneous melanoma.

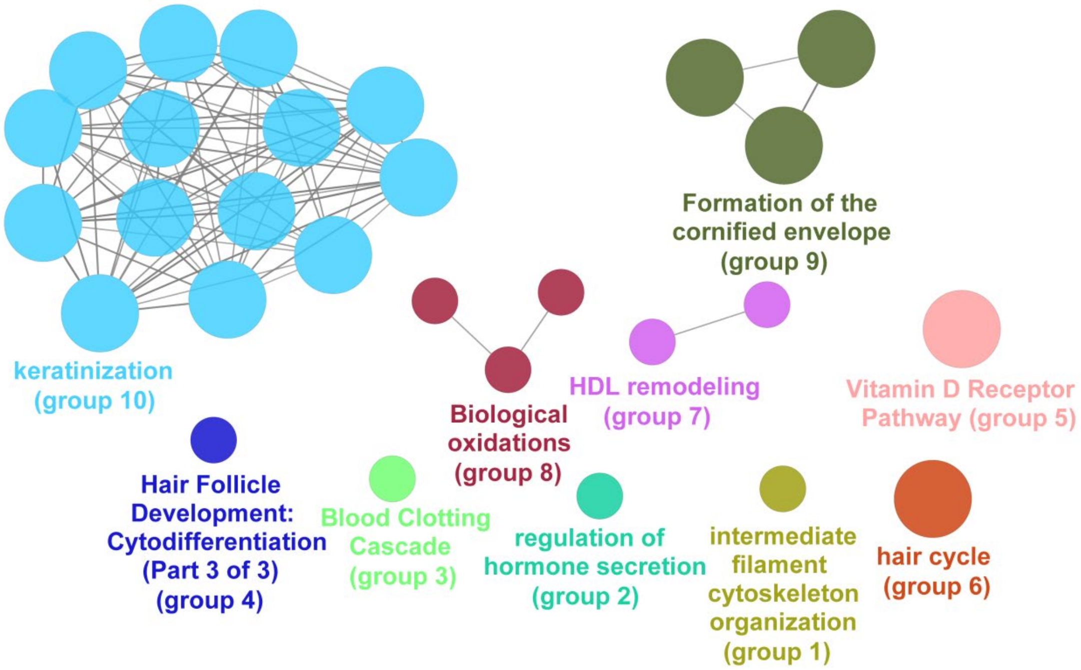

Figure S6. Grouped network of functional terms enriched in the downregulated genes in the immune_H tumor samples. Nodes represent the enriched terms (adjusted P-value<0.05). The size of the nodes reversely represents the statistical significance of the terms. Functionally related groups partially overlap. More detailed results were available in Table SIV.

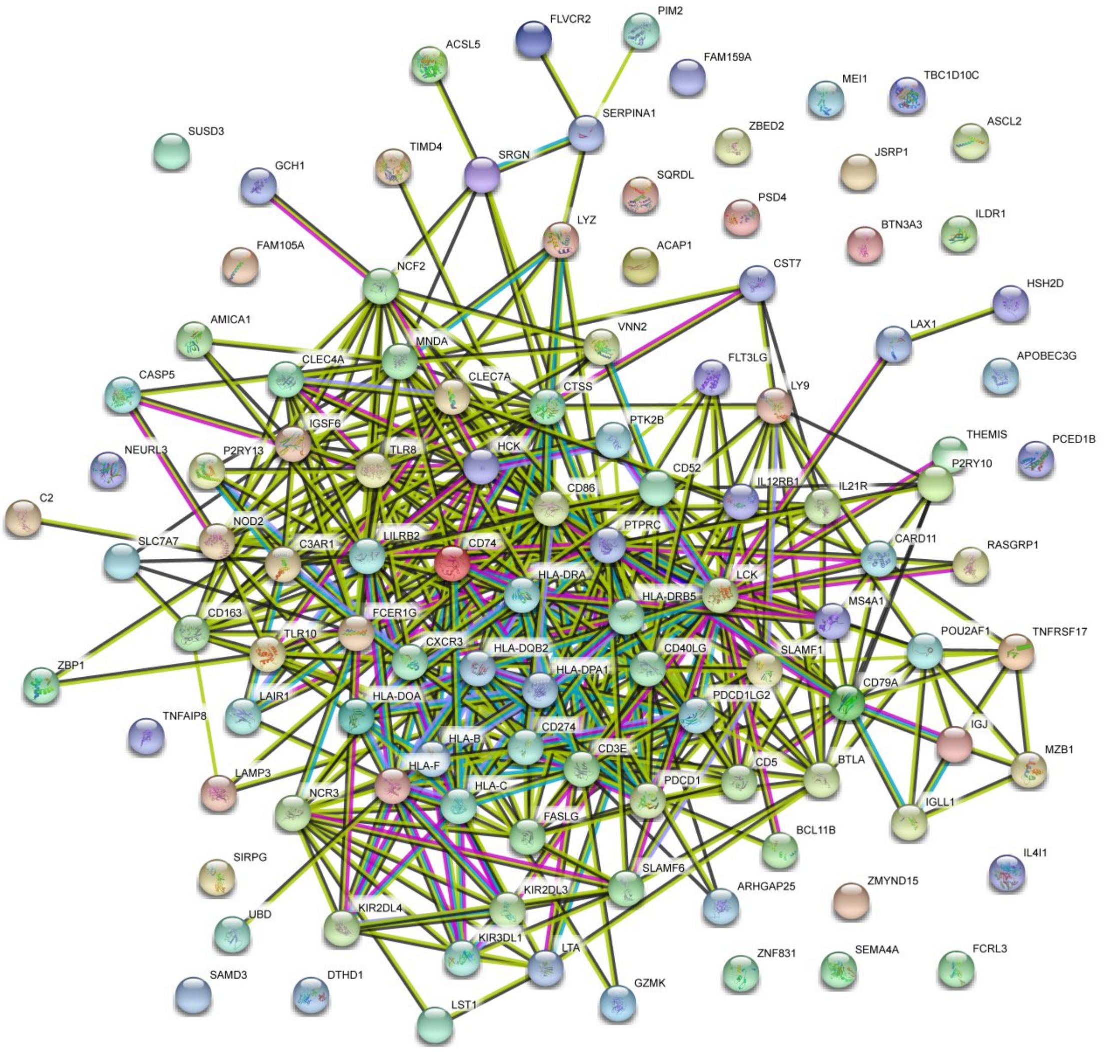

Figure S7. The PPI network for high-confidence prognostic genes. The network was generated by the web-based STRING database with default parameters (interaction score>0.4). 77.1% (84/109) of these genes had at least one neighbor protein. Colored nodes: query proteins and first of interactors. Empty nodes: proteins of unknown 3D structure. Filled nodes: some 3D structure is known or predicted. Edges represent protein-protein associations. Magenta represents experimentally determined, water-blue represents from curated database, green represents gene neighborhood, res represents gene fusions, dark blue represents gene co-occurrence, olive represents textmining, black represents co-expression and blue represents protein homology. PPI, protein-protein interaction; STRING, Search Tool for the Retrieval of Interacting Genes/Proteins.

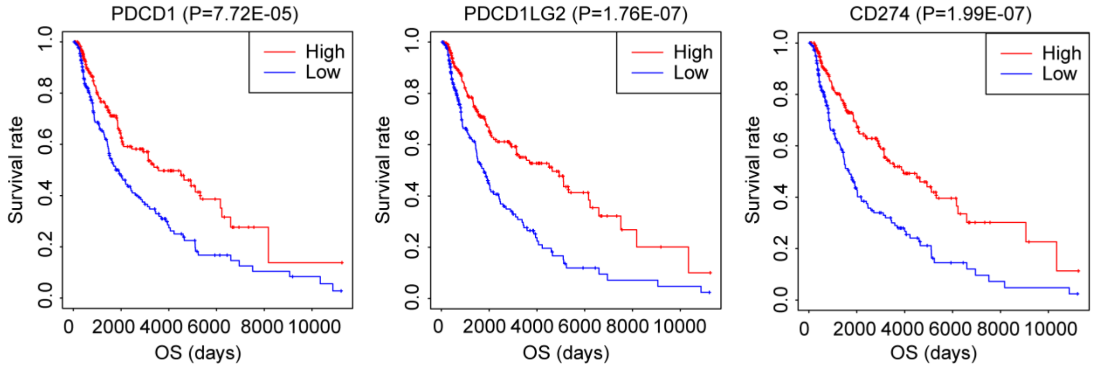

Figure S8. Kaplan-Meier OS curves of the immunomodulators from the identified gene module. The survival rates were estimated based on the TCGA dataset.
